# Epigenetic Age Estimation for Hawaiian False Killer Whales (*Pseudorca crassidens*) in the Absence of ‘Known‐Age’ Individuals

**DOI:** 10.1111/1755-0998.70099

**Published:** 2026-01-17

**Authors:** Karen K. Martien, Robin W. Baird, Kelly M. Robertson, Michaela A. Kratofil, Sabre D. Mahaffy, Kristi L. West, Susan J. Chivers, Frederick I. Archer

**Affiliations:** ^1^ Southwest Fisheries Science Center, National Marine Fisheries Service La Jolla California USA; ^2^ Cascadia Research Collective Olympia Washington USA; ^3^ Hawai’i Institute of Marine Biology Kāne‘ohe Hawai’i USA

**Keywords:** aging, cetaceans, DNA methylation, epigenetic clock, false killer whale, uncertainty

## Abstract

Epigenetic aging models hold great promise for enhancing many aspects of wildlife research and management. However, their utility is limited by the need to train models using known‐aged animals, which are rare among wildlife species. We present a novel approach to developing methylation‐based age prediction models that enables us to train models using samples from individuals whose chronological age is estimated with uncertainty based on photo‐identification catalogue data. Our approach incorporates this uncertainty into model training by representing the age of each individual with a probability distribution rather than a point estimate. We similarly represent the methylation profiles of individuals as binomial distributions and produce a distribution of predicted age for each sample that reflects the uncertainty in both its age and methylation profile. We compared age models trained using a wide range of parameterisations, training data sets and analytical methods to determine how well they predicted the catalogue‐based age estimates. The resulting model has a median absolute error of 1.70 years, outperforming many published clocks trained with known‐age samples. This approach significantly expands the range of species for which accurate methylation‐based age models can be developed, particularly those of conservation concern where known‐age samples are limited. By producing distributions of predicted age, it also enables researchers to accurately communicate the uncertainty in their age estimates to subsequent data users.

## 1 Introduction

Estimating age in wild populations has long been a challenge. Most species cannot be visually aged beyond assignment to general age categories (e.g., juvenile or adult). For species in which individuals are photographically identifiable or are marked (e.g., branding, tagging or genotyping) at birth, age can be determined through long‐term observational studies, though such an approach is time‐consuming and expensive. Most other methods (e.g., growth layer counts in otoliths, earplugs and teeth, aspartic acid racemisation in eye lenses, skeleto‐chronology) rely on having access to dead specimens, which has numerous drawbacks. First, it is exclusively backward looking, only providing age estimates for individuals that are no longer part of the population. Second, for species with remote distributions or large body sizes, it is extremely difficult, if not impossible, to amass a large collection of parts from dead animals. Yet, knowing the age of individuals can be hugely powerful for many types of studies, including estimation of life history and demographic parameters, epidemiological modelling, assessing trends in abundance and survivorship, evaluating parentage and social structure, and evaluating the impact of anthropogenic activities on populations (Doak and Morris 1999; Härkönen et al. 2007; Hernandez et al. 2023; Krahn et al. 2009; Photopoulou et al. 2017; Stepanuk et al. 2021).

Recent advances in genetics have led to the development of numerous Molecular Age Biomarkers (MABs), which can be used to estimate the age of individuals based on changes in features of DNA or RNA (Jarman et al. 2015). The molecular nature of these methods means that they can be used to estimate age from tissue samples collected from living animals, thereby eliminating many of the challenges associated with postmortem age estimation techniques. The most common molecular aging method is based on estimating the extent of methylation at specific cytosine residues (known as CpG sites) in an individual's genome. The proportion of genome copies that are methylated at certain CpG sites has been shown to either increase or decrease at a consistent rate through an individual's lifetime (Horvath 2013). Many CpG sites exhibit such ‘clock‐like’ behaviour across a broad range of mammalian taxa (Horvath 2013; Jarman et al. 2015; Polanowski et al. 2014). Methylation‐based age prediction models are developed by generating methylation profiles at a large set of known CpG sites for animals of known age and fitting a model that predicts age from a subset of the CpG sites. This approach has been used to develop methylation‐based age models for a wide range of wildlife species, including bats (Wilkinson et al. 2021), bears (Nakamura et al. 2023), elephants (Prado et al. 2021), equids (Larison et al. 2021), large cats (Qi et al. 2024), fishes (see review in Piferrer and Anastasiadi 2023), marine mammals (Barratclough et al. 2021, 2024; Beal et al. 2019; Bors et al. 2021; Hernandez et al. 2023; Mori et al. 2024; Parsons et al. 2023; Peters et al. 2022; Polanowski et al. 2014; Robeck et al. 2021, 2023) and birds (De Paoli‐Iseppi et al. 2017).

Though methylation‐based aging methods have dramatically expanded our ability to estimate ages of individuals in wild populations, there are several limitations to existing approaches that limit their applicability to many wildlife populations. Perhaps the greatest challenge is the need to create a predictive model using methylation data from a relatively large number of known‐age animals that span the full age distribution of the species. Simulations suggest that methylation‐based age prediction models should be trained using at least 70 known‐age samples, optimally 134 (Mayne et al. 2021), which are thresholds that studies rarely meet.

Methylation‐based aging models for wildlife species have been developed using samples from captive‐born individuals, whose ages are truly known (Barratclough et al. 2024; Larison et al. 2021; Robeck et al. 2023). In addition, the term ‘known‐age’ has also been applied to samples from individuals that were aged postmortem through traditional methods (Bors et al. 2021; Hernandez et al. 2023; Mori et al. 2024), and from individuals that were individually identified (e.g., in photographs from mark–recapture studies) the year they were born (Beal et al. 2019; Parsons et al. 2023; Peters et al. 2022; Polanowski et al. 2014), though in reality the ages of these samples are estimates that are presumed to be highly accurate. However, such data sets are not available for most wildlife species, particularly those of conservation and management interest.

Several approaches have been used in recent studies to address the lack of known‐age animals in many species. In studies involving limited numbers of known‐age samples of a particular species, researchers have developed multispecies clocks that combine samples for the focal species with those from other, often distantly related, species for which more samples were available. These studies have had mixed results. While several studies have succeeded in developing multispecies epigenetic age models with relatively low overall median absolute errors (Robeck et al. 2021, 2023; Wilkinson et al. 2021), subsequent studies have found that species‐specific clocks perform as well as or better than multi‐species clocks so long as sample sizes exceed 30–50 samples (Hernandez et al. 2023; Parsons et al. 2023; Peters et al. 2022). Thus, while multispecies, or ‘universal’ (Lu et al. 2023), clocks can be extremely valuable for species for which epigenetic age models cannot be developed, species‐specific clocks will be far more accurate when they can be developed (Parsons et al. 2023; Peters et al. 2022).

A second approach to expanding sample sizes in epigenetic age studies is to augment samples from known‐age individuals with samples from individuals whose age is estimated with some uncertainty. Researchers have accomplished this by classifying samples based on confidence in their age estimates and then either eliminating samples below a threshold value (Hernandez et al. 2023; Parsons et al. 2023; Prado et al. 2021) or down‐weighting samples with greater uncertainty (Barratclough et al. 2024; Peters et al. 2022). In the two studies that compared the performance of clocks trained with and without additional, higher‐uncertainty samples, the clock trained with the expanded data set performed worse than the one trained with fewer samples for which age uncertainty was low (Hernandez et al. 2023; Peters et al. 2022).

Kratofil et al. (2026) addressed a lack of samples from known‐age individuals by developing a protocol that uses data from a photo‐identification catalogue to estimate the age of individuals that are not typically photographically identifiable at birth. Their protocol combines quantitative metrics with qualitative information to estimate four values: the most likely age of the animal, a confidence rating for the most likely age estimate, and the minimum and maximum possible ages of the individual. They use these four values to generate an age probability distribution for each individual, which can be used in quantitative analyses that incorporate age information. Though Kratofil et al.'s (2026) approach has the potential to dramatically expand the number of species for which age estimates can be generated, there are currently no epigenetic age modelling approaches that can make use of age probability distributions.

Just as existing analytical methods cannot fully incorporate the uncertainty surrounding the age estimates of individuals, neither can they incorporate uncertainty in the estimates of individuals' methylation profiles. Uncertainty in the proportion of an individual's genome that is methylated at a given CpG site is a function of the number of sequencing reads generated at that site, with larger numbers of reads resulting in more precise estimates. Researchers typically account for uncertainty in methylation by setting a minimum read threshold and treating an individual's probability of methylation at a given CpG site as unknown if the threshold is not met (De Paoli‐Iseppi et al. 2017; Jarman et al. 2015; Zhou et al. 2018; Ziller et al. 2015). This approach can result in a large amount of data being thrown out and ignores uncertainty in the data that are retained.

We introduce a novel approach to developing methylation‐based age prediction models that explicitly incorporates uncertainty. Our method uses the age probability distributions from Kratofil et al. (2026) to represent each individual's age and binomial distributions to represent each individual's methylation profile. Our approach incorporates uncertainty from the input data and produces an estimated age probability distribution for each sample rather than a point estimate.

We demonstrate our approach by developing an age prediction model for Main Hawaiian Islands false killer whales ( *Pseudorca crassidens* ), which have been the subject of a long‐term photo‐identification study (Baird et al. 2008; Mahaffy et al. 2023). This small, resident population has experienced a dramatic, ongoing decline in abundance in recent decades (Badger et al. 2025; Bradford et al. 2018; Reeves et al. 2009) and was listed in 2012 as endangered under the US Endangered Species Act (Oleson et al. 2010). False killer whales are long‐lived, with a maximum lifespan of at least 65 years (Ferreira et al. 2014). Like most marine mammals, false killer whales are not born with natural markings that allow them to be individually identified, but rather become identifiable over time through the accumulation of nicks and notches on the dorsal fin (Baird et al. 2008; Mahaffy et al. 2023). Although the age of false killer whales can be estimated based on tooth growth layer groups (GLGs; Hohn et al. 1989; Hohn 2018), tooth‐based ages are only available from five animals that stranded in Hawai‘i between 2010 and 2016 (Kratofil et al. 2026). Thus, they are a prime example of a species of great management concern for which the standard epigenetic clock methods are inadequate.

## 2 Materials and Methods

### 2.1 Sample Selection and Catalogue‐Based Age Estimation

Our sample set consisted of 96 skin samples collected from 80 individuals. Most (*n* = 91) of the samples came from biopsies of live animals, while 5 were from animals that stranded dead. Of the 80 unique individuals that were sampled, 64 were each sampled once. The remaining 16 individuals were each sampled twice, between 2 and 13 years apart. All samples used in this study were part of the Southwest Fisheries Science Center's Marine Mammal and Sea Turtle Research Sample Collection (the MMaSTR Sample Collection), where they were either frozen or preserved in a 20% DMSO solution saturated with NaCl.

The chronological ages of all sampled animals at the time of sample collection were estimated by Kratofil et al. (2026) based on data from the Cascadia Research Collective's (CRC) long‐term photo‐identification catalogue. For each individual, Kratofil et al. estimated minimum (Age_min_), maximum (Age_max_), and ‘best’ (Age_best_) ages at the time of sampling, and a confidence rating (CR) on Age_best_. The values of CR range from 1 (very low confidence in Age_best_) to 5 (very high confidence). Age_min_ and Age_max_ were estimated to delimit the smallest range which one could be 100% confident encompassed the true age of the individual at the time it was sampled. We henceforth refer to these photo‐identification‐based age estimates collectively as the CRC age estimates.

Kratofil et al. (2026) generated age probability distributions for each individual by first estimating the location, scale and shape parameters of a Skew‐Normal distribution such that the mode of the distribution equaled Age_best_ and the 0.025 and 0.975 percentiles equalled Age_min_ and Age_max_, respectively. They then created a confidence‐weighted Skew‐Normal distribution for each individual that represented a weighted average of its Skew‐Normal and a Uniform distribution between Age_min_ and Age_max_. The weights used were 0.2, 0.55, 0.75, and 1.0 for animals with CR values of 2, 3, 4 and 5, respectively (Kratofil et al. 2026). These values were chosen by the researchers who estimated Age_best_ to reflect the level of uncertainty associated with each value of CR. As an example, the distribution for an individual with a CR of 2 would produce a scaled likelihood for a given age that is 80% influenced by a Uniform distribution (all ages between Age_min_ and Age_max_ being equally likely) and 20% influenced by that individual's Skew‐Normal distribution.

In addition to the CRC age estimates and age probability distributions, tooth growth layer group (GLG) age estimates were available (Kratofil et al. 2026) for the five samples from stranded animals. Because there are no probability distributions associated with the tooth GLG age estimates, we did not use them in the model training process. However, we compared the age estimates from our final epigenetic clock to the tooth GLG age estimates.

### 2.2 Laboratory Analyses

We used published data from a broad suite of taxa (De Paoli‐Iseppi et al. 2017; Grönniger et al. 2010; Hannum et al. 2013; Horvath 2013; Polanowski et al. 2014) to identify candidate CpG sites. We then used NCBI BLAST (Johnson et al. 2008) to find sites orthologous to 150 bp of sequence centred on each candidate CpG site in the published killer whale ( *Orcinus orca* ) genome (NCBI RefSeq GCF_937001465.1; Foote et al. 2022). Killer whales are the species most closely related to false killer whales for which a published genome was available at the time of primer design. From the killer whale genome we extracted sequences for 12 CpG‐rich fragments that have been shown to correlate with age in previous studies (Table S1 in Data S1). We used Zymo's Bisulfite Primer design tool (https://www.zymoresearch.com/pages/bisulfite‐primer‐seeker) to design bisulfite‐converted primers that amplify 263 to 400 bp amplicons for the extracted killer whale nuclear genes (Table S1 in Data S1).

We used targeted DNA methylation following the protocol of Masser et al. (2015) with minor changes, as noted below. Bisulifite conversion was done using Zymo's EZ DNA Methylation‐Lightning Kit (Zymo Research Co., Irvine, CA, USA) with 500 ng of gDNA in 20 μL. Two rounds of bisulfite conversion were performed in order to obtain sufficient bisulfite‐converted DNA for amplicon PCRs. We selected four samples for primer testing and optimisation, two from biopsies collected from animals in the wild and two from stranded animals to account for variation in sample quality. Final optimal annealing temperatures can be found in Table S1 in Data S1. We selected the eight loci that amplified most consistently for inclusion in our study.

Each bisulfite‐converted DNA sample was amplified for each locus per the optimal conditions for that locus, purified using SPRI beads (Agencourt AMPure XP, Beckman‐Coulter, Brea, CA, USA), and quantified using a Qubit and the Quant‐iT dsDNA high sensitivity Kit (ThermoFisher Scientific, Waltham, MA, USA). The 8 amplicons for each individual sample were then pooled by equal weight and volume. The library was prepared using the Nextera XT DNA Sample Preparation and Index Kits (Illumina, La Jolla, CA, USA) according to Masser et al. (2015). Three libraries were selected to check for average size of library peaks using the Bioanalyzer 2100 (Agilent Inc., Santa Clara, CA, USA). Quantification of the libraries was done using the Qubit and was subsequently normalised to 4 nM, pooled and sequenced on the Illumina MiSeq according to the manufacturer's instructions.

Most samples had been genetically sexed as part of previously published studies (Mahaffy et al. 2023; Martien et al. 2014, 2019). Those for which sex was not already available were genetically sexed using the assay described in Morin et al. (2005).

### 2.3 Estimating Methylation

We used the Galaxy web platform (Afgan et al. 2018) hosted at the public server usegalaxy.eu to align the sequence data. At each step of the data processing, we used FastQC (http://www.bioinformatics.babraham.ac.uk/projects/fastqc/) and MultiQC (Ewels et al. 2016) to assess data quality. We trimmed the reads using Trim Galore! (http://www.bioinformatics.babraham.ac.uk/projects/trim_galore/), which was designed for use with bisulfite‐converted reads. FastQC revealed consistently low nucleotide diversity at the 5′ ends of all sequences, so we removed nine bp from the 5′ end of each sequence.

We aligned the trimmed reads to the bisulfite‐converted target sequences using Bismark (Krueger and Andrews 2011), then removed duplicate reads using Bismark's Deduplicate function and used SAMtools (Li et al. 2009) to generate a pileup file for each sample at each locus. Finally, we used iVar (Grubaugh et al. 2019) to generate consensus sequences from the pileup files and compared the consensus sequences for each locus to the killer whale sequences used to design primers.

We summarised the reads at all CpG sites and at all sites in our reference sequences that were cytosines (Cs) not followed by guanine (referred to as non‐CpG cytosine sites). For each CpG and non‐CpG cytosine site in each sample we counted the frequency of each nucleotide (A, C, G or T), as well as the total number of reads. At CpG sites, the reads containing Cs represent methylated reads (*n*_m_), while at non‐CpG cytosines they represent reads that were not converted to thymines (Ts) during bisulfite conversion (though see Fuso 2018). At both types of sites, we considered reads containing As or Gs to be sequencing errors. We therefore calculated the corrected coverage (*K*_m_) at each site by subtracting the reads containing As or Gs from the total number of reads. We estimated the bisulfite conversion efficiency for each sample ($P_{v_{i}}$) by dividing the total number of Ts mapped to all non‐CpG cytosine sites (*n*_v_), which represent correctly converted reads, by the sum of the corrected coverage at those sites (*K*_v_) (Masser et al. 2015). We calculated the sequencing error rate by dividing the number of As and Gs at all cytosine sites by the total number of reads at those sites.

We first calculated the observed probability of methylation (*P*_*m*_) at each CpG site (*j*) in each sample (*i*) as follows:

(1) $$P_{m_{i,j}}=\frac{n_{m_{i,j}}}{K_{m_{i,j}}}.$$

We calculated the true probability of methylation ($\hat{P_{m}}$) by correcting for the bisulfite conversion efficiency for the *i*‐th individual ($P_{v_{i}}$), as described in Masser et al. (2015):

(2) $$\hat{P_{m_{i,j}}}=1-\left(\frac{1-P_{m_{i,j}}}{P_{v_{i}}}\right).$$

In cases where *P*_*v*_ ≤ (1−*P*_*m*_), which resulted in $\hat{P_{m}}$ being less than or equal to 0, we set $\hat{P_{m}}$ equal to one half the lowest value of $\hat{P_{m}}$ for that locus. We used logit($\hat{P_{m}}$) as our estimate of methylation, to ensure that methylation was being modelled on an additive scale as several of the models we examined were linear in nature.

We examined correlations in our estimates of methylation among CpG sites from the same locus by calculating Pearson's correlation coefficients and their associated *p* values. We also calculated Pearson's correlation coefficients between each CpG site and Age_best_ and sex. All calculations and statistical analyses were conducted using custom scripts in the statistical programming language R (v4.4.1; R Development Core Team 2022) and are available from GitHub (https://github.com/kmartien/FKW_epigenetic_age).

### 2.4 Model Design

**FIGURE 1:**
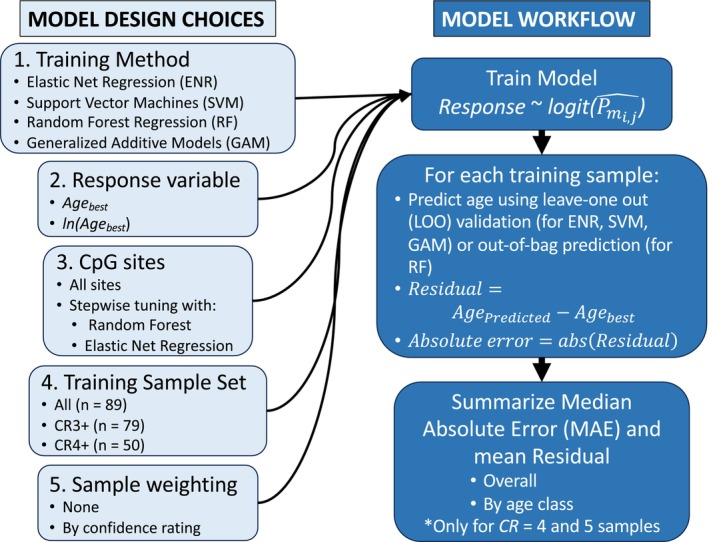
Design choices and workflow for training candidate age prediction models. CR3+ refers to all samples with confidence rating (CR) of 3 or higher, while CR4+ refers to samples with CR 4 or higher. *P*_mi,j_ is the probability of methylation of the *i*th individual at the *j*th CpG site. Regardless of what samples were used to train a model, the median absolute error (MAE) and mean residual were summarised only for the samples with CR = 4 and 5.

We developed multiple models that use our methylation data to predict the CRC age estimates. We evaluate five model design choices (Figure 1): (1) the method used to train the model, (2) whether to train models to predict Age_best_ or ln (Age_best_), (3) the CpG sites to include in the model, (4) the samples used in the training sample set, and (5) weighting of samples in the training sample set based on confidence rating. For all methods except those trained with Random Forest Regression (RFR), we used leave‐one‐out (LOO) cross‐validation to evaluate the predictive accuracy of the resulting models. Because a random subset of samples is left as out‐of‐bag in each tree in a RFR model, we directly used the out‐of‐bag age predictions to assess the predictive accuracy of models trained with RFR.

For all predictions, we recorded the residual for each individual as the difference between the predicted age and Age_best_, and the deviance as the absolute value of the residual. We used the median deviance as our primary metric of each model's predictive accuracy. For consistency with previous publications, we henceforth refer to the median deviance as the median absolute error (MAE). Differences between Age_best_ and predicted age can either be the result of poor predictive power of a model or discrepancies between Age_best_ and actual chronological age. Therefore, when assessing the predictive power of the age prediction models, we calculate MAE only for samples with confidence ratings of 4 or 5.

#### 2.4.1 Training Method

We test four different methods for training models: elastic net regression (ENR; Friedman et al. 2010), RFR (Breiman 2001), support vector machines (SVM; Cortes and Vapnik 1995) and generalised additive models (GAM; Wood 2017). ENR, the most commonly employed analytical approach used for training methylation‐based age prediction models, is limited to linear regression when applied to continuous explanatory variables, such as estimates of methylation (Friedman et al. 2010). SVM is similarly restricted to linear functional forms (Cortes and Vapnik 1995), but was found in one recent study to perform better than ENR (Qi et al. 2024). RFR, in contrast, is a nonparametric approach, while GAM can fit a different functional form for every CpG site, including nonlinear models.

For the ENR models, we used the *cv.glmnet*() function of the package *glmnet* v4.1–8 (Friedman et al. 2010) in the statistical modelling language R v4.4.1 to build models using 10‐fold cross‐validation. The parameter *alpha* controls the blend between ridge regression, which tends to retain correlated predictors but minimises their coefficients, and LASSO regression, which tends to select a single predictor from a group of correlated predictors. The standard approach to optimising the value of alpha is to run *cv.glmnet()* one time for each value of alpha from 0.1 to 0.9 at intervals of 0.1, and choose the value of alpha that minimises the median absolute error (MAE). However, we found that the difference in MAE between values of *alpha* was often of similar magnitude to the variability in MAE between replicate runs of *cv.glmnet()* at a given value of *alpha*. Furthermore, for some of our models MAE was minimised at values of alpha below 0.1 or above 0.9. Therefore, we used the *optim()* function to choose the optimum value of alpha for each ENR model in a way that accounts for variability between replicate runs. For each value of *alpha*, we conducted 1000 replicate runs of *cv.glmnet()*, recorded the MAE at *lambda.min*, and calculated median(MAE) across replicates. We evaluated values of *alpha* between 0.0001 and 0.5 and chose the value with the lowest median(MAE).

We fit RFR models using the R package *randomForest* v4.7–1.1 (Breiman 2001; Liaw and Wiener 2002). To tune each model, we constructed RFR models across a grid of two parameters, *sampsize* (the number of samples randomly drawn for each tree in the forest) and *mtry* (the number of sites randomly drawn at each node in a tree). The grid was constructed with all combinations of integers from 0.3n to 0.7n for *sampsize* and all integers from 0.1s to 0.5s for *mtry*, where *n* and *s* are the number of samples and CpG sites used to train the model, respectively. We selected as the optimum model the combination of *sampsize* and *mtry* that had the minimum out‐of‐bag mean square error based on a visual inspection of the mean square error distribution across the parameter space. All RFR models were grown with 10,000 trees. Convergence of all models was assessed by inspecting the trace of out‐of‐bag mean square error across all trees.

We used the R package *e1071* v1.7–16 (Meyer et al. 2024) to build prediction models using SVM (Cortes and Vapnik 1995). We used the *tune.svm()* function to select the best values of the *cost* and *gamma* parameters of a radial basis function (*rbf*) kernel. The tuning used 10‐fold cross validation to search a grid spaced every 0.1 over the range 10^−4^ to 10^5^ for *cost* and 10^−5^ to 10^4^ for *gamma*.

We fit GAMs using the R package *mgcv* v1.9–1 (Wood 2023). The smoothing basis predictor for each site was a thin plate regression spline with a smoothing penalty. We set the dimensionality of the bases (the *k* parameter in the *mgcv::te()* function) at three. If the model did not converge, we incremented *k* by one until convergence. We used the *predict.gam()* function within the *mgcv* package to predict ages.

#### 2.4.2 CpG Site Selection

In addition to training models using all CpG sites, we used a stepwise tuning approach in which we first selected CpG sites that were informative in predicting age and then trained age prediction models using only those sites. A similar approach resulted in improved final model performance in a previous epigenetic age model study (Qi et al. 2024). We identified informative sites using ENR or RFR. For site selection with ENR, we conducted 1000 replicate regression runs using all samples in the training data set (see below) and selected sites that were included in the final model in at least 50% of the replicate runs. We refer to the selected sites as the ENR sites. For RFR, we used the by‐site permutation‐based percent increase in mean square error metric (Liaw and Wiener 2002) to evaluate site importance and estimated the significance of the importance scores with 1000 permutations using the R package *rfPermute* v.2.5.3 (Archer 2024). We again included all samples in the site‐selection regressions. We selected sites with significance *p* values less than 0.05 and refer to them as the RFsites.

#### 2.4.3 Training Sample Selection and Weighting

In order to evaluate the trade‐offs between maximising sample size in the training data set and excluding samples for which the estimate of Age_best_ is highly uncertain, we developed the age prediction models using all samples, only samples with a confidence rating on Age_best_ of 3 and higher (henceforth called CR3+ samples), and only samples with a confidence rating of 4 and higher (CR4+ samples).

We trained models with samples unweighted and with samples weighted by their value of CR. ENR and GAM use sample weights when calculating the fit of a set of regression coefficients, while RFR uses weights when selecting samples to include in each tree, resulting in highly weighted samples being included in the trees more often than those with low weights. SVM does not have a mechanism for weighting samples.

### 2.5 Resampling

We used resampling to incorporate uncertainty in age and uncertainty in methylation into our age predictions. For each resampled model, 1000 random replicates were drawn for all of the data, with a separate model fit to each replicate. In each replicate, the age for each sample was derived from a random draw from the confidence‐weighted Skew‐Normal age probability distribution described by Kratofil et al. (2026) for that sample, and the methylation profiles were calculated by replacing the point estimate of probability of methylation for each individual at each CpG site (Equation 1) with a random deviate from a binomial distribution:

(3) $$P_{m_{i,j,k}}\sim \text{Binom}\left(n_{m_{i,j}},K_{m_{i,j}}\right)$$

A separate model was fit to each replicate, and age was predicted for every sample within each replicate as described above, except that if an age was predicted to be less than zero or greater than 80 years old, the prediction was set to either of these respective boundaries to represent that the probability of estimates outside these boundaries is zero. Though the oldest estimated age for a false killer whale is 65 years (Ferreira et al. 2014), we set the boundary higher because it is unlikely that the maximum observed age represents the maximum possible age. We used the *hdi()* function in the R package *HDInterval* (v0.2.4; Meredith and Kruschke 2022) to calculate the 95% highest density interval (HDI) of predicted age across all replicates for each sample. We calculated the proportion of samples for which Age_best_ fell within the HDI.

We also used the resampling replicates from pairs of samples taken from the same individual at different timepoints to predict the age difference between the two samples, as well as their age ordinality (i.e., which sample in a pair is older). We recorded the difference between the predicted age estimates of the samples in each pair in each replicate and calculated the proportion of replicates in which the ordinality was correctly determined.

## 3 Results

### 3.1 Sample and Locus Selection

From the 12 primer pairs we designed, we chose the eight that performed best during optimisation to include in our study. The loci we chose contained a combined total of 207 CpG sites and 2289 bp of sequence per sample (Table S1 in Data S1). The consensus sequences for four of the loci were identical to the sequences we extracted from the killer whale genome, while the remaining four differed by one or two substitutions (loci GRIA2, HMG20B and PRDM12) or a single deletion (locus FLJ0945) (Table S1 in Data S1). The primer sequences, extracted killer whale sequences, and consensus false killer whale sequences are provided in Supporting Information.

In order to eliminate poor performing samples and CpG sites for which estimates of methylation are likely to be highly uncertain, we first removed from our data set six samples whose median read depth across all CpG sites was less than 1000 reads. We then eliminated 23 CpG sites whose median read depth across the remaining samples was less than 1000 reads. Finally, we eliminated one sample that had fewer than 100 reads at one of the remaining CpG sites.

**FIGURE 2:**
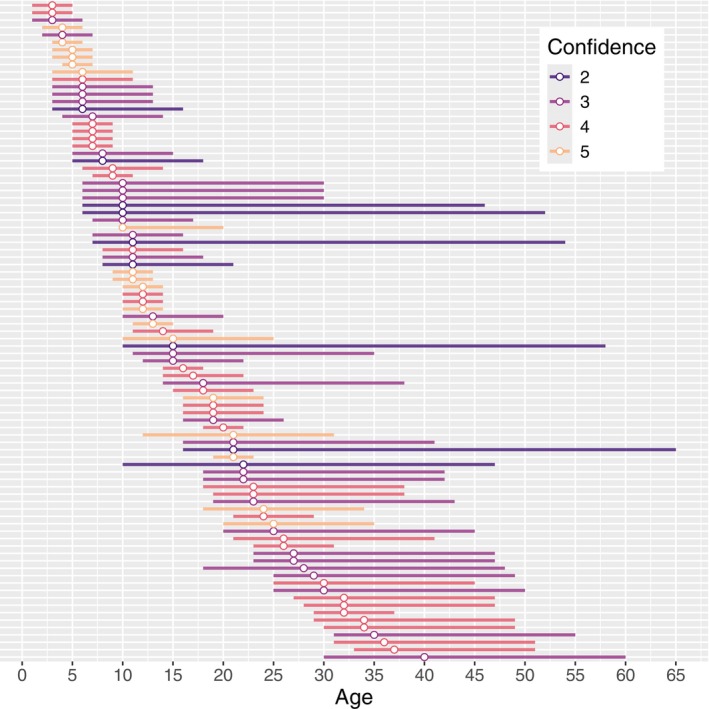
Distribution of CRC ages for all 89 samples. Length of line ranges from minimum to maximum age. Point is Age_best_ and colour represents confidence rating.

The final data set consisted of 89 samples (40 female, 49 male) from 76 individuals (36 female, 40 male) (Table 1, Figure 2, Figure S1 in Data S1), including 13 pairs of samples taken from the same animal between 2 and 13 years apart. Estimates of Age_best_ ranged from 3 to 40 years. Median read depth across CpG sites for the remaining samples ranged from 1103 to 6435 reads, while median read depth per CpG site across individuals ranged from 1006 to 7375 reads (Figure S2 in Data S1). Bisulfite conversion efficiency was high, ranging from 0.993 to 0.997 across samples. Conversion efficiency was consistent across amplicons. Sequencing errors were low, constituting only 0.02% of all reads.

**TABLE 1:** Details on samples used in this study. Estimates of Age_best_ are based on catalogue‐based age estimates from Kratofil et al. (2026). Age bins are aligned with age class breaks used by Kratofil et al.

Confidence rating	Sample size	Sample size by age bin			Median Age_best_	Minimum Age_best_	Maximum Age_best_
		0–9	10–24	25–40			
2	9	2	7	0	11	6	22
3	30	7	15	8	15	3	40
4	32	9	13	10	18.5	3	37
5	18	6	11	1	11.5	4	25
All	89	24	46	19	14	3	40

**FIGURE 3:**
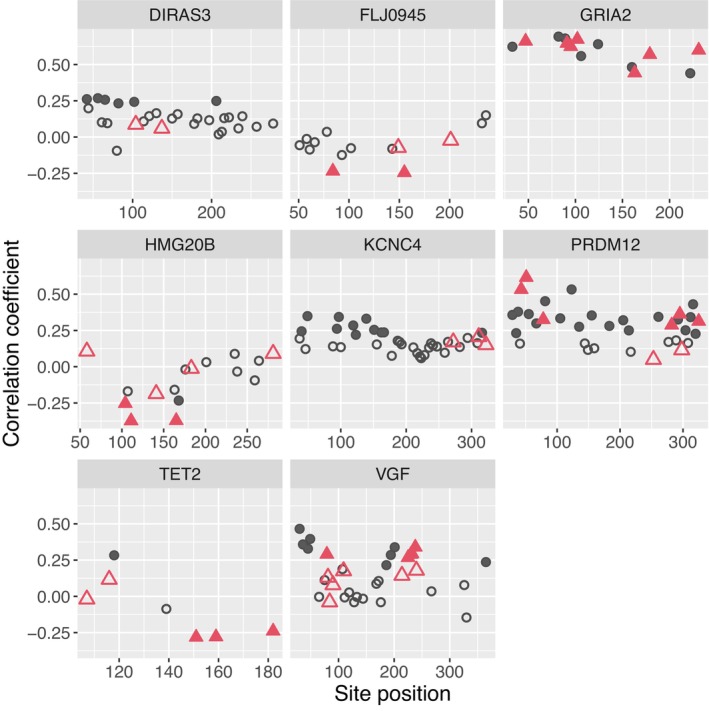
Pearson's correlation coefficients between methylation and Age_best_ for each CpG site on each locus. Sites with positive coefficients exhibit increasing methylation with age, while those with negative coefficiens exhibit decreasing methylation with age. Sites that were included in the final false killer whale age clock are shown with red triangles, while all other sites are shown with grey circles. Correlation coefficients that are significantly different from zero (*p*‐value ≤ 0.05) are represented by filled symbols, while non‐significant correlation coefficients are represented by open symbols.

The extent to which methylation was correlated among CpG sites from the same locus varied across loci. One‐third or more of all pairwise correlation coefficients among CpG sites is greater than 0.7 for the loci DIRAS3, GRIA2, KCNC4 and TET2, while the remaining loci exhibited few to no correlation coefficients among CpG sites above 0.5 (Figure S3 in Data S1). Eighty‐three (83) of the 184 CpG sites are significantly correlated with Age_best_ (*p*‐value ≤ 0.05), though only 15 have correlation coefficients with absolute value greater than 0.5 (filled symbols on Figure 3). Plots of Age_best_ versus the probability of methylation ($\hat{P_{m}}$) for each CpG site are provided in supplemental material (Figure S1 in Data S1).

### 3.2 Model Design

**FIGURE 4:**
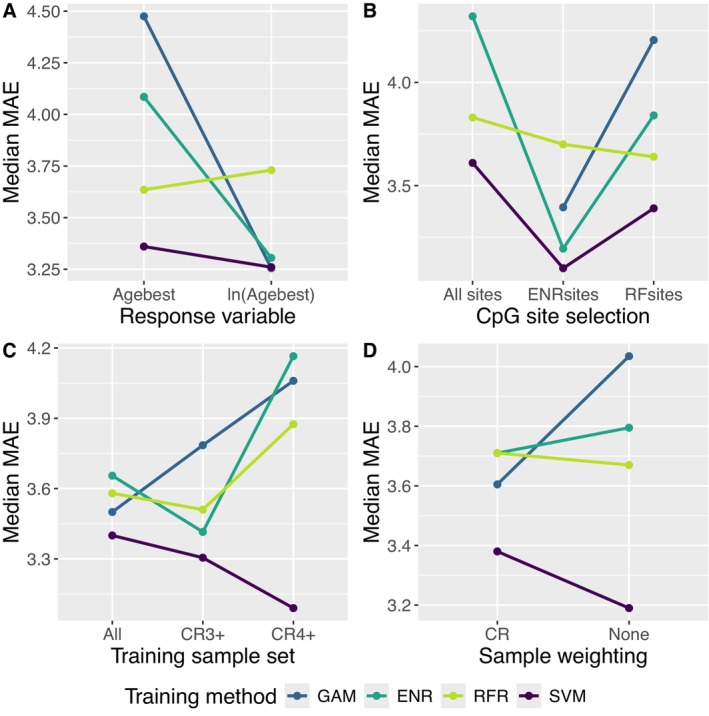
Summary of the prediction accuracy of models trained by each analytical method as a function of different model design parameters. Each point represents the median absolute error (MAE) value for all models trained using a given combination of training method and a given value for one other model design parameter. The panels show the relative performance of models based on (A) whether or not Age_best_ was log‐transformed, (B) which CpG sites were used to train the models, (C) which samples were used to train the models, and (D) whether samples were weighted based on CR. Lines connect points representing median performance of models trained with the same training method to highlight the change in median performance that resulted from changing each model design parameter. Training methods are generalised additive model (GAM), elastic net regression (ENR), random forest regression (RFR), and support vector machine (SVM).

SVM consistently produced models with the lowest MAE (Figure 4; Table S2 in Data S1). The impact of other model design choices varied depending on what method was used to train models. Log‐transforming Age_best_ tended to significantly reduce MAE for ENR and GAM, but had little impact for SVM and RFR (Figure 4A). For all model training methods, lower MAEs were achieved when CpG sites were selected through stepwise tuning (Figure 4B). However, SVM, GAM and ENR performed best when sites were selected by ENR, while RFR performed best when Random Forest was used for site selection. Finally, ENR, RFR and GAM models tended to have lower MAEs when all or most (CR3+) samples were included and were weighted by CR, while SVM performed best with only high CR (4 and 5) samples and no sample weighting (Figure 4C,D). Note that while SVM does not have a mechanism for weighting samples, the performance of models trained with SVM varied depending on whether or not samples were weighted during stepwise tuning.

### 3.3 Resampling

When we used resampling to incorporate uncertainty in age and methylation into age prediction models, the proportion of samples for which the 95% HDI of the predicted age probability distribution included Age_best_ ranged from 0.5 to 0.96 (Table 2). The distributions of predicted ages produced by GAM models were the most consistent and generally the most accurate, with their HDIs including Age_best_ for 76%–96% of the samples, while the models produced by RFR only resulted in HDIs that included Age_best_ for 50%–66% of the samples. Results for ENR and SVM were more variable, ranging from 68%–94% for ENR and 64%–90% for SVM.

**TABLE 2:** Predictive accuracy of the 5 models with the lowest overall median absolute error (MAE) for each training method (ENR = elastic net regression; SVM = support vector machines; GAM = generalised additive models; RF = random forest regression).

Model rank	Training method	Training samples	Weight	Age transform	Site selection method	Site selection samples	Corr	MAE				Mean				Age_best_ in 95% HDI
								Overall	0–9 year	10–24 year	25+ year	Overall	0–9 year	10–24 year	25+ year	
1	SVM	CR4+	No	None	ENR	CR4+	0.93	1.70	1.44	1.50	3.00	0.11	1.65	0.86	−3.62	0.90
2	GAM	CR4+	Yes	Log	ENR	All	0.82	2.04	1.58	1.86	9.44	−0.31	1.59	0.33	−4.30	0.94
3	GAM	CR4+	No	Log	ENR	CR3+	0.79	2.18	1.18	1.73	5.96	0.84	2.61	−0.30	0.91	0.96
4	ENR	CR3+	Yes	Log	ENR	All	0.83	2.20	1.45	2.06	7.62	−1.19	1.42	−0.15	−7.05	0.74
5	ENR	CR3+	Yes	Log	ENR	CR3+	0.86	2.23	1.41	2.54	5.19	−1.02	1.48	−0.64	−5.24	0.76
6	ENR	CR4+	No	None	ENR	CR4+	0.91	2.25	1.34	1.85	4.12	0.03	1.54	0.64	−3.38	0.94
7	ENR	CR4+	Yes	Log	ENR	CR3+	0.85	2.25	1.97	2.36	4.91	−0.41	1.84	−0.53	−3.23	0.82
8	SVM	CR4+	Yes	None	ENR	All	0.84	2.28	1.37	2.28	6.45	−0.20	2.12	1.46	−6.97	0.64
9	SVM	CR4+	Yes	Log	ENR	CR4+	0.84	2.33	0.93	1.92	6.10	0.06	2.44	0.82	−4.83	0.72
10	ENR	CR3+	No	Log	ENR	CR3+	0.84	2.36	1.47	2.67	7.64	−1.19	1.76	−0.59	−6.55	0.68
11	SVM	CR4+	Yes	None	ENR	CR3+	0.9	2.39	2.18	1.95	5.30	−0.04	1.28	1.14	−4.41	0.72
13	GAM	CR4+	No	Log	ENR	CR4+	0.84	2.47	1.45	2.26	6.37	−0.32	1.91	0.49	−5.12	0.92
15	SVM	CR4+	No	None	RFR	CR3+	0.84	2.53	2.32	2.49	6.26	−0.40	1.79	1.31	−7.10	0.74
20	GAM	All	No	Log	ENR	CR4+	0.79	2.61	1.26	2.60	7.93	−1.66	1.59	−0.52	−8.58	0.76
27	GAM	CR3+	No	Log	ENR	All	0.74	2.72	1.96	2.80	9.08	−1.48	1.49	0.01	−8.81	0.80
57	RFR	CR3+	No	Log	RFR	CR4+	0.78	3.02	1.12	2.86	7.71	−1.33	2.62	−0.43	−8.69	0.54
60	RFR	CR3+	No	None	RFR	CR3+	0.78	3.05	1.69	2.73	6.48	−0.16	3.49	0.80	−7.24	0.54
63	RFR	All	Yes	None	ENR	All	0.8	3.07	1.27	2.65	6.25	−0.34	3.26	0.66	−7.43	0.66
64	RFR	CR3+	Yes	None	RFR	CR4+	0.77	3.07	1.49	2.88	5.62	−0.10	3.63	0.96	−7.49	0.50
73	RFR	CR3+	No	None	RFR	All	0.78	3.13	1.71	2.86	6.31	−0.15	3.52	0.83	−7.30	0.52

*Note:* The MAE and mean residual are given for all samples, as well as for those with Age_best_ estimates between 0 and 9 years, 10 to 24 years, and 25 to 40 years. Corr is the Pearson's correlation coefficient between Age_best_ and predicted age. ‘Age_best_ in 95% HDI’ gives the proportion of the confidence rating 4 and 5 samples for which the 95% high density interval (HDI) of the predicted age probability distribution from resampling encompassed Age_best_. Models are sorted by overall MAE.

### 3.4 False Killer Whale Age Clock

**FIGURE 5:**
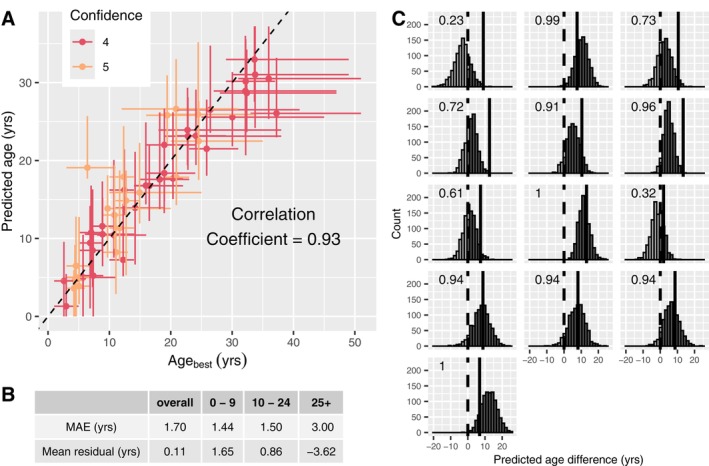
Predictive accuracy of the top‐ranked model, now termed the false killer whale age clock. The model was trained by SVM using CpG sites selected through stepwise tuning with ENR. Both the stepwise tuning model and the final model were trained using only samples with confidence ratings of 4 or 5 (CR4+ samples). Samples were unweighted. (A) Age_best_ versus predicted age for the training samples. Horizontal bars span from Age_min_ to Age_max_, vertical bars span from the lower to the upper value of the 95% high density interval (HDI) from resampling, and points are colour coded by the confidence rating on Age_best_ (red = confidence 4, orange = confidence 5). (B) Table showing median absolute error (MAE) and mean residual overall and by age group. (C) histograms of the differences in predicted ages from the resampled models of pairs of samples taken from the same individual in different years. The difference is calculated as the predicted age of the older sample minus the predicted age of the younger sample, resulting in a positive difference (shown in dark grey bars) if the ordinality of the sample ages is correctly predicted and a negative difference (light grey bars) if ordinality is incorrectly predicted. The proportion of the histogram that is greater than zero, and therefore depicts correct ordinality, is shown on each panel. The solid vertical lines show the actual differences in the sample ages (calculated as the difference in their collection dates), while the dotted lines are at zero.

The age prediction model that had the best overall performance was one trained using SVM with CpG sites chosen through stepwise tuning with ENR, and with no log‐transformation of estimates of Age_best_ (Table 2). Both the site selection ENR model and the final SVM model were trained with only the CR4+ samples with no sample weighting. The model had an overall MAE (based on the LOO predictions) for the training samples of 1.70 years, mean residual of 0.11 years, and a correlation between Age_best_ and predicted age of 0.93 (Figure 5A). It included 47 CpG sites (red triangles on Figure 3). We chose this model as the false killer whale age clock. Here we report the results from the LOO cross‐validation estimates for the CR4+ samples used to train the clock, as well as the age predictions for the CR = 2 and 3 samples when the methylation profiles of those samples were analysed with the clock.

The LOO age estimates for the CR4+ samples are more precise for younger samples. We calculated the median absolute age error and the mean residual within three age groups that were chosen to correspond to breaks between the subadult/adult (10 years) and adult/physically mature adult (25 years) age classes defined by Kratofil et al. (2026). MAE is 1.44 and 1.50 years for samples with Age_best_ less than 10 years, and between 10 and 24 years, respectively. However, MAE increases to 3.00 years for samples with Age_best_ of 25 years and higher (Figure 5B). There is a slight tendency for the model to overestimate the ages of young samples and underestimate the ages of older samples, with mean residuals of 1.65 years, 0.86 years, and −3.62 years for the three age groups, respectively (Figure 5B). There was no difference in the predictive accuracy of the model between sexes (MAE = 2.92 and 2.91 for females and males, respectively).

The predicted age probability distributions produced for the training samples when we used resampling to incorporate uncertainty into the analysis included Age_best_ within the 95% HDI for 90% (45 out of 50) of the samples. The predicted age probability distributions also correctly identified the older sample for 11 out of 13 pairs of samples taken from the same individual in different years (Figure 5C). For eight of the pairs, the older sample had a higher predicted age in greater than 90% of resampling replicates, while in three pairs, the older sample had a higher predicted age in 61% to 73% of replicates. For the two pairs for which the ordinality was not correctly predicted, the older sample had a higher predicted age in only 23% and 32% of replicates (Figure 5C).

GLG counts from IsoMet saw‐prepared teeth are available for four of the five samples in our training data set that were collected from stranded animals (Kratofil et al. 2026). The residual between the clock predicted ages of these samples and their GLG counts ranged from −2.62 to 4.61, with a mean of 0.85 (Table 3). The HDI for each sample was wider than the range of GLG counts but narrower than the range of plausible ages (Age_min_ to Age_max_) from the catalogue‐based estimates of Kratofil et al. (2026). In all cases, the mean and high GLG counts were within the HDI, though for two samples the low GLG count was outside the HDI. GLG counts are also available from coral saw‐prepared teeth for all five stranded animals (Kratofil et al. 2026). However, Kratofil et al. reported difficulty visualising the central layers of those teeth, rendering their counts less reliable than the IsoMet saw‐prepared teeth.

**TABLE 3:** Comparison of age estimates for four samples for which tooth growth layer group estimates (GLG count) from IsoMet saw‐prepared teeth are available (Kratofil et al. 2026).

Field ID	IsoMet saw mean GLG count (low–high)	Age_best_ (Age_min_–Age_max_)	Predicted age (HDI min–max)
KW2010019	25 (22–28)	24 (20–34)	26.32 (23.15–35.18)
KW2013018	22 (20–25)	25 (20–35)	22.09 (17.52–27.66)
KW2015015	22 (18–24)	21 (17–31)	26.61 (20.77–33.02)
KW2016020	16 (14–20)	10 (6–20)	13.38 (7.54–18.11)

*Note:* Each tooth was read three times, with the results presented as the mean of the three counts, the lowest count, and the highest count. Age_best_, Age_min_, and Age_max_ are catalogue‐based estimates from Kratofil et al. (2026). Predicted age is the leave‐one‐out prediction from the final false killer whale age clock, with the minimum and maximum values of the high density interval (HDI) given in parentheses. The first three columns of the table are reproduced from Kratofil et al. (2026) with permission. Kratofil et al. also provide GLG counts from coral saw‐prepared teeth from these four individuals, plus one additional stranded animal that is included in our data set. However, because Kratofil et al. reported difficulty visualising the growth layers of the coral saw‐prepared teeth, we do not consider those counts here.

**FIGURE 6:**
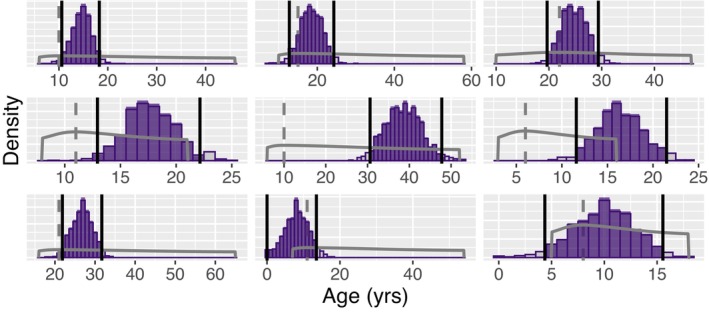
Predicted age probability distributions from the false killer whale age clock for samples with confidence ratings of 2. Each panel shows results for a different individual. The solid black lines show the lower and upper limits of the 95% high‐density interval (HDI) of the distribution. Values outside of the HDI are shown in grey bars. The dashed grey line is at Age_best_, while the solid grey lines show the Skew‐Normal age probability distribution from Kratofil et al. (2026).

When we used the clock to predict the ages of the samples with CR = 2 and 3, the differences between Age_best_ and predicted age were larger than for the CR4+ samples, as expected. Given the higher uncertainty in the estimates of Age_best_ for the CR = 2 and 3 samples, differences between Age_best_ and predicted age could be due to discrepancies between Age_best_ and chronological age, inaccuracy of the clock prediction, or a combination of both. For all of the CR = 2 samples (Figure 6) and most (24 out of 30) of the CR = 3 samples (Figure S5 in Data S1), the width of the predicted age probability distribution HDI was less than the range of plausible ages (Age_min_ to Age_max_) estimated from catalogue‐based data (Kratofil et al. 2026), indicating lower uncertainty surrounding the clock‐based age estimate as compared to the catalogue‐based age estimate.

## 4 Discussion

The methylation‐based epigenetic age clock model we developed for Main Hawaiian Island false killer whales predicted the catalogue‐based age estimates with an overall MAE of 1.70 years, despite being trained using samples with a much higher degree of age uncertainty than is conventional. Our approach produces distributions of predicted age for each individual in the data set by incorporating uncertainty in the input data, thereby dramatically expanding the range of species for which the development of methylation‐based age models is possible.

All studies using data from ‘known‐age’ wild‐born individuals have some uncertainty in the ages of training samples. Previously published epigenetic age studies of cetaceans have typically dealt with this by restricting the training data set to individuals with chronological age known with high precision, typically within 1 year (Parsons et al. 2023; Polanowski et al. 2014; Robeck et al. 2023). In contrast, for the CR4+ samples used to train our age clock, the difference between the youngest (Age_min_) and oldest (Age_max_) possible age for each sample ranged from 3 to 20 years (Figure 2 and Figure S1 in Data S1). Despite this uncertainty, the resulting model is able to predict the point estimates of age (Age_best_) with greater precision than many previously developed epigenetic age clocks (e.g., Barratclough et al. 2024, MAE = 1.91; Hernandez et al. 2023, MAE = 2.09; Parsons et al. 2023, MAE = 2.26–3.73; Peters et al. 2022, MAE = 2.1).

It is important to note that our model is actually predicting the results of the CRC age estimation process, rather than true age. Though we believe these two values to be highly correlated, they are not necessarily the same. As described in Kratofil et al. (2026), the CRC process is very detailed and incorporates a wide range of factors that encompass the extent of CRC's sighting history with each animal. The results of this process are likely the best description of our knowledge of age for free‐swimming Hawaiian false killer whales. However, for many samples, the uncertainty in age is quite large (Figure 2). The value of our resampling approach is that it can carry this age uncertainty (along with the uncertainty of the methylation process) forward and both are reflected in the uncertainty of the prediction results. Furthermore, the resampling we describe is not restricted to the confidence‐weighted Skew‐Normal age probability distributions generated by Kratofil et al.'s age estimation method. Rather, researchers can choose the distribution that best describes age uncertainty in their data set. For data‐poor populations, this could be as simple as a Uniform distribution between estimates of minimum and maximum possible age for each individual.

Though it is typically not incorporated in down‐stream analyses, there is some degree of uncertainty even in ages derived from conventional methods (Zhang et al. 2024; Zhao et al. 2019). For example, age estimates from growth layers in teeth, otoliths, and earplugs can vary between readers as well as between readings of the same sample for the same reader (Hohn and Fernandez 1999; Osborne et al. 2022). Furthermore, the accuracy of age estimates derived from growth layer counts is known to vary with the age class (Barratclough et al. 2023; Osborne et al. 2022). While conventional aging methods may provide sufficient resolution for many applications, researchers should consider uncertainty when modelling age data. Our model structure also provides a way to incorporate age estimates ranging from those that are relatively unknown to those that are known with high precision in the same model.

Our results highlight the importance of testing different model training methods and model design parameters when developing epigenetic age clocks. We found that the impact of different model design choices was dependent on the method used to train a given model. For instance, restricting the training sample set improved performance for models trained with SVM but reduced performance for those trained with GAM. For all training methods, performance was improved when CpG sites were first chosen through stepwise tuning. However, models trained by RFR performed best when the stepwise tuning used RFR, while the other training methods performed best when ENR was used for stepwise tuning. These specific interactions between model design parameters may not be generalise to other species or data sets. Consequently, systematic testing of combinations of model design parameters should be a standard component of epigenetic age clock development.

Our clock tends to underestimate the ages of older individuals, a pattern that has been observed in other studies (Barratclough et al. 2024; Parsons et al. 2023; Peters et al. 2022). There are several possible explanations for the decreased predictive accuracy of methylation‐based age clocks at older ages. Sample sizes tend to decrease with estimated age, both because relatively few individuals survive to older ages and because more data (e.g., longer sighting history; Kratofil et al. 2026) are often required to estimate age in older individuals. None of our samples with CRC age estimates of 25 years or more had confidence ratings of 5, and 44% had confidence ratings < 4. When we included samples with confidence ratings < 4 in the training data set, prediction accuracy generally declined, confirming that the increase in the sample size from older individuals was outweighed by the increased imprecision of their age estimates (see also Hernandez et al. 2023; Peters et al. 2022). Prediction accuracy of older individuals could likely be improved by including more samples with both older (> 25 years) age estimates and high confidence in those estimates, but such samples are likely to be difficult to obtain. Our results highlight the need for future studies to include estimates of both the median absolute age error and the mean residual of their models as a function of age class, as well as measures of uncertainty in individual age estimates. These values will allow for more accurate interpretation of the age estimates produced when models are applied to samples of unknown age.

The difficulty in obtaining samples from older individuals with high‐confidence age estimates likely contributes to the fact that weighting samples by CR generally did not improve performance of models. Because age estimates for older individuals tend to have greater uncertainty than those of young individuals, weighting will tend to disproportionately down‐weight samples from older age classes, exacerbating the problem of small sample sizes from and contributing to the higher error rates for older age classes. Nonetheless, it is possible that weighting would be of value in other studies or that a different weighting scheme would result in improved performance for our data set.

For the four samples with tooth GLG counts, the clock age estimates closely matched the GLG counts, with a mean residual of 0.85 (Table 3). The mean residual between the clock age estimates and the CRC estimates for these samples is 2.1, which is higher than the mean residual for samples with Age_best_ of 10 to 24 (mean residual = 0.85) or ≥ 25 (mean residual = −3.62). Given the small number of samples for which there are GLG counts, it is difficult to draw firm conclusions from this discrepancy. However, it is worth noting that the samples with tooth GLG estimates are the only samples in our sample set from stranded animals. Barratclough et al. (2024) found that methylation‐based age estimates tended to overestimate chronological age in samples from bottlenose dolphins with low scores on a scale designed to represent an individual's 1 year survival probability, as well as in individuals that were directly impacted by the Deep Water Horizon oil spill. It is possible that the higher mean residuals for the samples with GLG counts compared to those without reflects more rapid physiological ageing in animals that strand dead compared to those that are live‐biopsied. Further sampling of stranded individuals may help to resolve this source of uncertainty.

Both ENR and SVM are restricted to linear functional forms (Cortes and Vapnik 1995; Friedman et al. 2010). However, the relationship between age and methylation may not be linear at some CpG sites. Nonlinear functional forms, such as asymptotic or exponential, could result in differences in predictive power as a function of age and could therefore contribute to the greater error observed for older individuals in many methylation‐based age prediction models (Barratclough et al. 2024). Many studies use log or square root transformations of age in an attempt to correct for the nonlinearity that results from accelerated physiological aging that occurs in the years prior to sexual maturation (Horvath 2013; Peters et al. 2022). We found that log transformation substantially improved predictive accuracy for models trained with ENR but had little impact on those trained with SVM (Figure 4A). RFR, in contrast, is a non‐parametric approach and therefore may be better able to account for nonlinear relationships between age and methylation. However, because it cannot extrapolate beyond the values in the training set, it can also result in greater prediction errors for older individuals due to the smaller sample sizes that are typically available in older age classes (Barratclough et al. 2024; Parsons et al. 2023). GAM is the only method we examined that can explicitly accommodate non‐linear functional forms. It is possible that the performance of models trained with GAM could be improved if stepwise tuning were conducted using an analytical method that is also able to account for nonlinear relationships.

The ability to accurately estimate age in wildlife species is a vital component of conservation and management. By developing a resampling approach that explicitly incorporates input uncertainty, we have demonstrated that accurate age models can be built even for species for which traditional ‘known‐age’ samples are not available. Our results further underscore the importance of systematic testing of model design decisions to optimise the performance of an epigenetic age clock. This framework effectively removes a major barrier to the development of epigenetic clocks for many species of conservation concern, facilitating the integration of age data into broader wildlife management strategies.

## Author Contributions

**Karen K. Martien:** conceptualisation, data curation, formal analysis, methodology, visualisation, writing – original draft preparation, writing – review and editing. **Robin W. Baird:** conceptualisation, data curation, funding acquisition, writing – review and editing. **Kelly M. Robertson:** data curation, investigation, writing – review and editing. **Michaela A. Kratofil:** data curation, methodology, writing – review and editing. **Sabre D. Mahaffy:** data curation, methodology, writing – review and editing. **Kristi L. West:** data curation, writing – review and editing. **Susan J. Chivers:** data curation, writing – review and editing. **Frederick I. Archer:** formal analysis, methodology, visualisation, writing – original draft preparation, writing – review and editing.

## Funding

This work was supported by the US Commander Pacific Fleet Environmental Readiness Division, Dolphin Quest, Cascadia Research Collective and National Marine Fisheries Service.

## Conflicts of Interest

The authors declare no conflicts of interest.

## Supporting information


**Data S1:** men70099‐sup‐0001‐DataS1.zip.
**Table S1:** Primer design and optimization details for the 12 loci for which we designed primers. The first eight loci were used in this study, while the remaining four were eliminated following optimisation. Start and end positions and sequence length refer to the total sequence extracted from the *O. orca* genome for use in primer design, while amplicon length is length of the product amplified by the primers we designed. The final column lists the number of mutations between the *O. orca* sequence and the false killer whale consensus sequence generated from our data (for loci retained in study) or optimisation notes (for loci omitted).
**Table S2:** Predictive accuracy of all models (*n* = 318). The MAE and mean residual are given for all samples, as well as for those with Age_best_ estimates between 0 and 9 years, 10 to 24 years, and 25 to 40 years. Corr is the Pearson's correlation coefficient between Age_best_ and predicted age. Models are sorted by overall MAE.
**Figure S1:** The difference between Age_max_ and Age_min_ as a function of Age_best_. Points are colour‐coded based on their confidence rating. Note that, for a given age class, the range of plausible ages for high‐confidence samples (confidence ≥ 4) is consistently smaller than for low‐confidence samples.
**Figure S2:** Median number of reads per sample (A) and per CpG site (B) in the final data set.
**Figure S3:** Heat maps showing absolute value of correlation coefficients of methylation among CpG sites within a locus.
**Figure S5:** Predicted age probability distributions from the false killer whale age clock for samples with confidence ratings of 3. Each panel shows results for a different individual. The solid black lines show the lower and upper limits of the 95% high‐density interval (HDI) of the distribution. Values outside of the HDI are shown in grey bars. The dotted grey line is at Age_best_, while the solid grey lines show the Skew‐Normal age probability distribution from Kratofil et al. (2026).
**Figure S4:** Plots of Agebest versus the probability of methylation ($\hat{P_{m}}$) for all 184 CpG sites included in the final data set. Each plot is labeled with [locus name]_[position]. The line of best fit, as calculated using the R function geom_smooth(method = ‘lm’), is shown in blue.

## Data Availability

All data and code used in this study are freely available on GitHub (https://github.com/kmartien/epigenetic_age_with_uncertainty).
