# Supplementary material for: Epigenetic Age Estimation for Hawaiian False Killer Whales (Pseudorca crassidens) in the Absence of ‘Known‐Age’ Individuals
Source: Mol Ecol Resour. 2026 Jan 17;26(2):e70099. doi: 10.1111/1755-0998.70099 (PMC12811820; doi:10.1111/1755-0998.70099)
Supplement: Supplementary file 1 — Data S1: men70099‐sup‐0001‐DataS1.zip. Table S1: Primer design and optimization details for the 12 loci for which we designed primers. The first eight loci were used in this study, while the remaining four were eliminated following optimisation. Start and end positions and sequence length refer to the total sequence extracted from the O. orca genome for use in primer design, while amplicon length is length of the product amplified by the primers we designed. The final column lists the number of mutations between the O. orca sequence and the false killer whale consensus sequence generated from our data (for loci retained in study) or optimisation notes (for loci omitted). Table S2: Predictive accuracy of all models (n = 318). The MAE and mean residual are given for all samples, as well as for those with Agebest estimates between 0 and 9 years, 10 to 24 years, and 25 to 40 years. Corr is the Pearson's correlation coefficient between Agebest and predicted age. Models are sorted by overall MAE. Figure S1: The difference between Agemax and Agemin as a function of Agebest. Points are colour‐coded based on their confidence rating. Note that, for a given age class, the range of plausible ages for high‐confidence samples (confidence ≥ 4) is consistently smaller than for low‐confidence samples. Figure S2: Median number of reads per sample (A) and per CpG site (B) in the final data set. Figure S3: Heat maps showing absolute value of correlation coefficients of methylation among CpG sites within a locus. Figure S5: Predicted age probability distributions from the false killer whale age clock for samples with confidence ratings of 3. Each panel shows results for a different individual. The solid black lines show the lower and upper limits of the 95% high‐density interval (HDI) of the distribution. Values outside of the HDI are shown in grey bars. The dotted grey line is at Agebest, while the solid grey lines show the Skew‐Normal age probability distribution from Kratofil et al. [file MEN-26-e70099-s001.zip › men70099-sup-0001-DataS1/men70099-sup-0001-supinfo.docx]

**Supplemental Information for:**

**Epigenetic age estimation for Hawaiian false killer whales**

**(*Pseudorca crassidens*) in the absence of ‘known age’ individuals**

Karen K. Martien

Robin W. Baird

Kelly M. Robertson

Michaela A. Kratofil

Sabre D. Mahaffy

Kristi L. West

Susan J. Chivers

Frederick I. Archer

Table. S1. Primer design and optimization details for the 12 loci for which we designed primers. The first eight loci were used in this study, while the remaining four were eliminated following optimization. Start and end positions and sequence length refer to the total sequence extracted from the O. orca genome for use in primer design, while amplicon length is length of the product amplified by the primers we designed. The final column lists the number of mutations between the O. orca sequence and the false killer whale consensus sequence generated from our data (for loci retained in study) or optimization notes (for loci omitted).

| Locus | Reference | | O. orca chromosome | Start position | End position | Sequence length | Forward Tm | Reverse Tm | Final annealing temp | Amplicon length | # CpG sites | Substitutions relative to O. orca |
| --- | --- | --- | --- | --- | --- | --- | --- | --- | --- | --- | --- | --- |
| TET2 | Grönniger et al 2010 | | NW_004438445.1 | 6543392 | 6543750 | 358 | 55.9 | 58.4 | 57 | 192 | 10 | 0 |
| GRIA2 | Chakrabarti et al 2001, Koch et al 2011 | | NW_004438421.1 | 18635500 | 18635950 | 450 | 61.9 | 57.9 | 58 | 243 | 15 | 2 |
| DIRAS3 | Horvath 2013 | | NW_004438546.1 | 4692750 | 4693250 | 500 | 55.6 | 55.4 | 54 | 278 | 28 | 0 |
| FLJ0945 | Horvath 2013 | | NW_004438525.1 | 7905350 | 7905850 | 500 | 55.2 | 55.4 | 54 | 285 | 20 | 2-bp deletion in FKW |
| KCNC4 | Horvath 2013 | | NW_004438417.1 | 25124695 | 25125250 | 555 | 60.7 | 57.8 | 58 | 325 | 40 | 0 |
| VGF | Horvath 2013 | | NW_004438442.1 | 15154650 | 15155220 | 570 | 55.9 | 56.5 | 55 | 348 | 35 | 0 |
| HMG20B | Horvath 2013 | | NW_004438719.1 | 477160 | 477777 | 617 | 58 | 57.6 | 57 | 265 | 19 | 1 |
| PRDM12 | Horvath 2013 | | NW_004438443.1 | 2072900 | 2073400 | 500 | 64.3 | 65 | 64 | 353 | 40 | 1 |
| Loci not chosen: | |  |  |  |  |  |  |  |  |  |  |  |
| Locus | Reference | | O. orca chromosome | Start position | End position | Sequence length | Forward Tm | Reverse Tm |  | Amplicon length | # CpG sites | Notes |
| KHDRBS2 | Horvath 2013 | | NW_004438437.1 | 19776130 | 19776679 | 549 | 57.6 | 56.1 |  | 324 | 9 | Inconsistent amplification |
| CDKN | Koch et al 2011 | | NW_004438482.1 | 622300 | 622800 | 500 | 59.7 | 58.8 |  | 340 | 41 | Inconsistent amplification |
| KLF14 | Horvath 2013 | | NW_004438464.1 | 12005300 | 12005869 | 569 | 56.3 | 55.4 |  | 279 | 30 | Inconsistent amplification |
| SCGN | Horvath 2013 | | NW_004438470.1 | 858233 | 858813 | 580 | 55.7 | 57.1 |  | 343 | 20 |  |

Table S2. Predictive accuracy of all models (n = 318). The MAE and mean residual are given for all samples, as well as for those with *Age_best_* estimates between 0 and 9 years, 10 to 24 years, and 25 to 40 years. Corr is the Pearson’s correlation coefficient between *Age_best_* and predicted age. Models are sorted by overall MAE.

Table in separate Excel spreadsheet


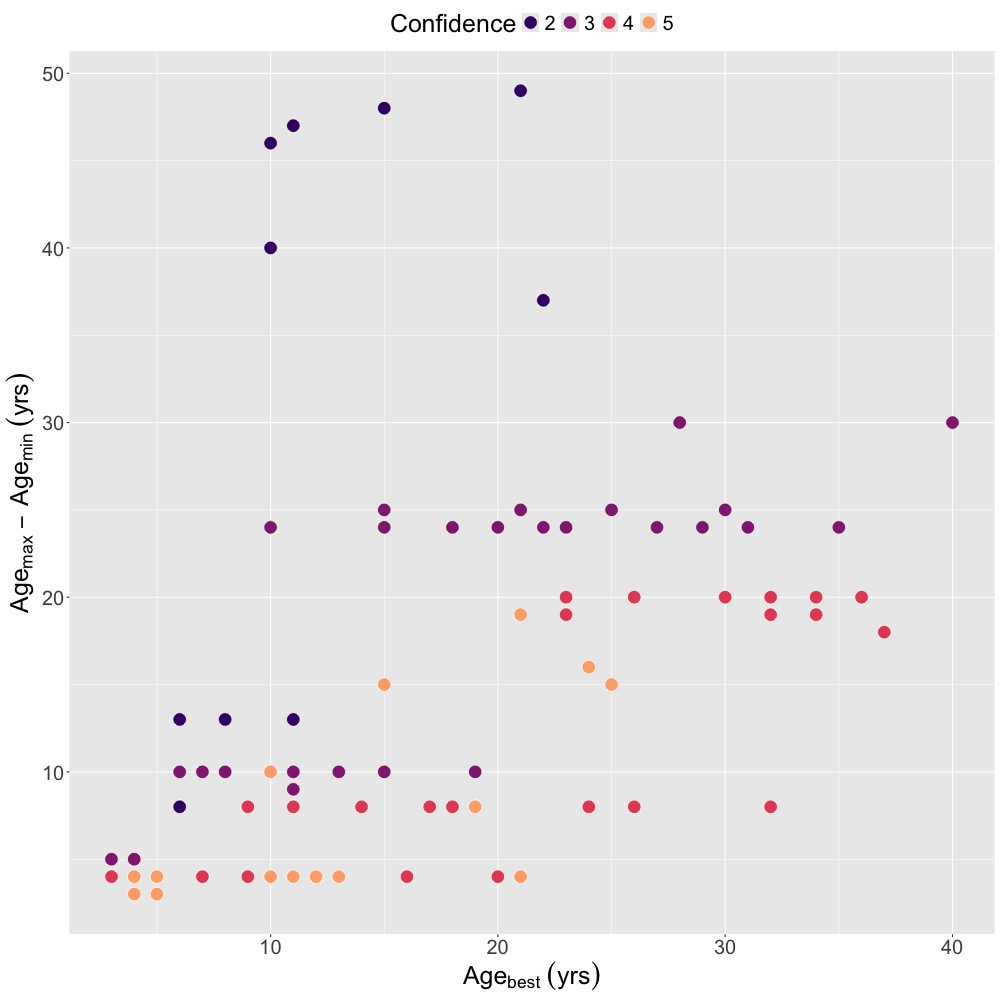


Figure S1. The difference between *Age_max_* and *Age_min_* as a function of *Age_best_*. Points are color-coded based on their confidence rating. Note that, for a given age class, the range of plausible ages for high-confidence samples (confidence ≥4) is consistently smaller than for low-confidence samples.


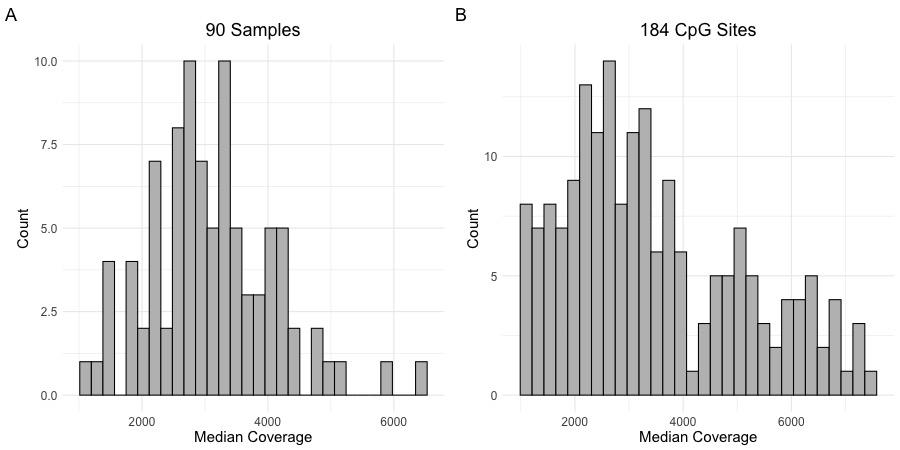


Figure S2. Median number of reads per sample (A) and per CpG site (B) in the final data set.


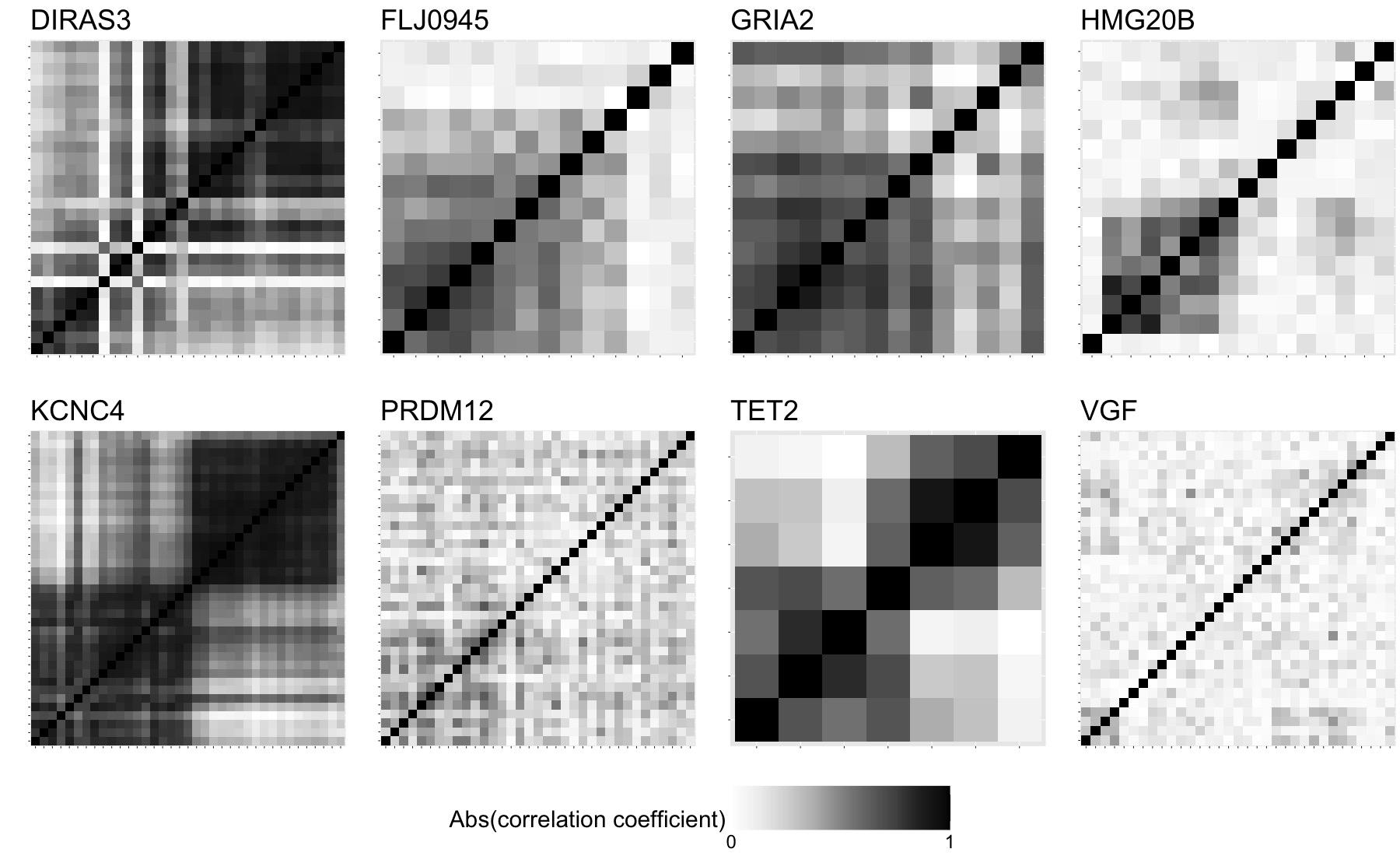


Figure S3. Heat maps showing absolute value of correlation coefficients of methylation among CpG sites within a locus.


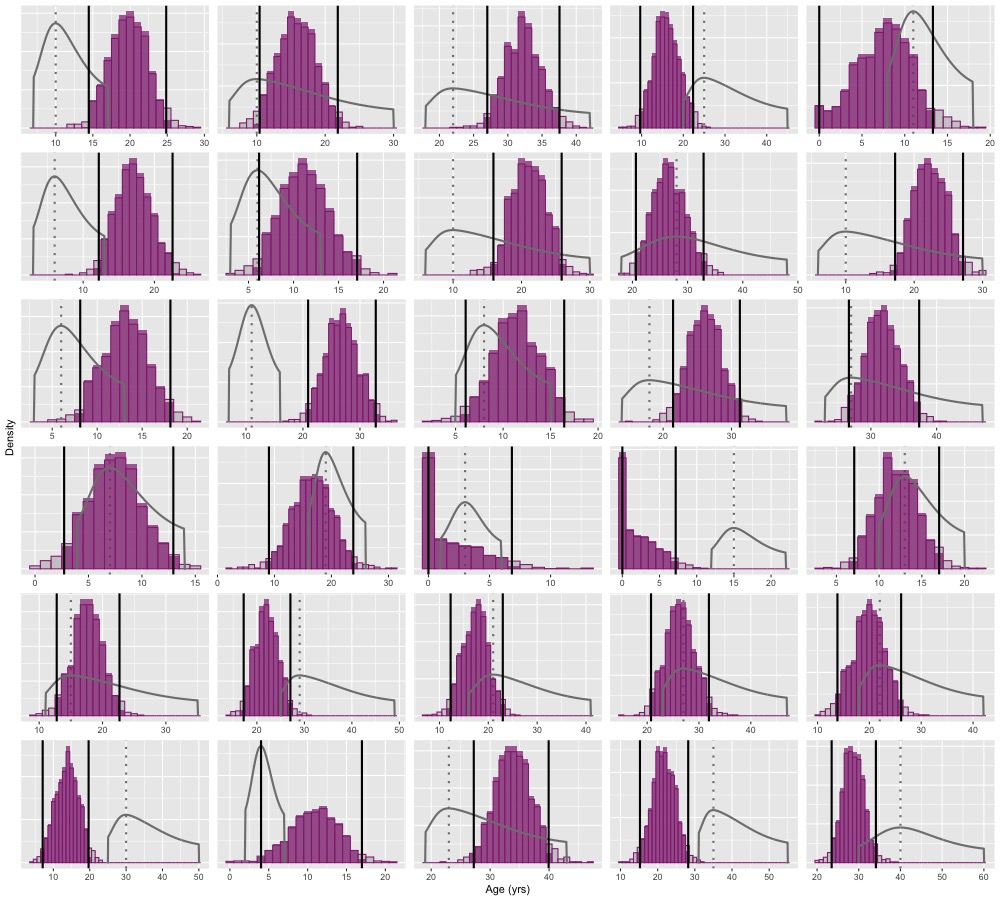


Figure S5. Predicted age probability distributions from the false killer whale age clock for samples with confidence ratings of 3. Each panel shows results for a different individual. The solid black lines show the lower and upper limits of the 95% high-density interval (HDI) of the distribution. Values outside of the HDI are shown in gray bars. The dotted gray line is at *Age_best_*, while the solid gray lines show the Skew-Normal age probability distribution from Kratofil et al. (2026).

Primer Sequences:

>TET2_F1

TGGGTTGATGTTATTATTTAATTATAAAGTTGG

>TET2_F2

TGGGTCGATGTTATTATTTAATTATAAAGTTGG

>GRIA2_F1

GAGTGTATGGGAGGGTGTTGAGTGTG

>DIRAS3_F1

TTATTTGTATTTGTTTGTTTTAGAGGAGG

>DIRAS3_F2

TTATTCGTATTTGTTTGTTTTAGAGGAGG

>FLJ0945_F1

TTGTTTAATTTAAATATGGAAATGTTTAGGTTAGG

>KCNC4_F1

GGTGTTGTTGGTAGGTTATTTAGTAGTAGGG

>KCNC4_F2

GGTGTTGTCGGTAGGTTATTTAGTAGTAGGG

>VGF_F1

TTAGTGGGTTGGGTTTAGTTGG

>HMG20B_F1

TTTTTTAGAGTTTTTTTTTGAGGGAGGATTTAGG

>PRDM12_F1

AGGGGTTGTAGGGAGGAGGGG

>PRDM12_F2

AGGGGTCGTAGGGAGGAGGGG

>TET2_R1

TTTTAAAAACTCAACAAATTAAAAATTTAACCCAAACC

>TET2_R2

TTTTAAAAACTCGACAAATTAAAAATTTAACCCAAACC

>GRIA2_R1

TTTCCAAAAAAAATAAAACATCCACAAAATAC

>DIRAS3_R1

TTTAAATTAACAAATCAACTCAAAAAAAAACTTC

>DIRAS3_R2

TTTAAATTAACGAATCAACTCAAAAAAAAACTTC

>FLJ0945_R1

ATACCAAAAAAAAATAAAACCCAAATACCC

>KCNC4_R1

CCTCCATCCAAAACATATCTAAAAAAAAAAATAAC

>KCNC4_R2

CCTCCGTCCAAAACATATCTAAAAAAAAAAATAAC

>VGF_R1

CATTCTACCATTCATTCATTCATCCTTTTC

>HMG20B_R1

AAACAAAACCTCAACATCTAAAAAAAAATCTAAAC

>HMG20B_R2

AAACGAAACCTCAACATCTAAAAAAAAATCTAAAC

>PRDM12_R1

TCCTCATCCTCCCCCATCACAACC

>PRDM12_R2

TCCTCGTCCTCCCCCATCACAACC

Killer whale locus sequences:

>TET2-Oorc-wPrimer

TGGGTCGATGTCATTACTTAATTATAAAGCTGGACATTTTTTCAAAATAGAGGCTCTTATGGGGGTAAATTTAAGCATTTGAAAGTGCAGATTTATTTCTTACAACCGAATCCAGCGCGAAGTGGAGAGTCACTGGTGCGAAAGCCCTCTCGGATCTGCGTGAATCCTTCTTCCCAGCCCACGTGGCCAAAGTAAACAGAAGGTGGGCCGGGGCGGGGAGAAGCGGGCCTGGGTCAAATTCCTAATTTGTCGAGTCTTTAAAA

>GRIA2-Oorc-wPrimer

GAGTGCATGGGAGGGTGCTGAGTGTGCCGAGGCGCTGGGACCACAGCGGCAGCCTGCTGGAAGCTGCATCCAGCCAGTCTCCGGACTTCGCGAGCGGGGACCGGGCGCAGGAAGGCAGCCACCCGCAGGACCTTGGAAATAGGGATTCCTCTGCCACCACGTCGGGTTTCTAGCAGCTCGGTGCTACATTGCTGTCTCAAAATGCAGAGGATCTAATTTGCCGAGGAGAACGGCCAAAGAAGGAAGAGGAGGAAAAGGGGGGAAAAAAAGGGTATTTTGTGGATGCTCTACTTTTCTTGGAAA

>DIRAS3-Oorc-wPrimer

TTATTCGTATTTGTTTGTTTTAGAGGAGGAGTAGAGATTTTCGCGTGGTGGTGTTCGGTTCGGTCGGCGTGGGTAAGAGCGCGTTGGTGTAGAGGTGGGTGCGCGGTAATTTTCGTGAGGCGTATTTGTCGATTATCGAAGATATTTATCGTTAGGCGTTGGGTTGTAGTTATAAGGCGGGCGTATTGTATATTATCGATATTATCGGCGGTCGTCGTTATCGGGGTTTGTAGCGTTTCGTTATTGTTAGGGGTTACGTTTTTATTTTGGTTTATTTCGTTATTAAGAAGTAAATTTTGGAGGAGTTGAAGTTTTTTTTTGAGTTGATTCGTTAATTTAAA

>FLJ0945-Oorc-wPrimer

CTGCCCAATTTAAATATGGAAATGCCCAGGCCAGGCAATGCAATTATTCCCGAGGCCCGGCGGGCCGTGCAGGCCCTCGGCTTCGGGGAGCACGGTGGCATCGCCCCCTTTCTGGTGTGTGTGAAATTATGTGCACAAAAGGCGTCTCCGTATCCGAGTGTGTGAACTAAATAAATAAATAAGGGCTTTTGTTTGTTTGCCGGCTCCTGCACATGACTGTTTTGACTAAACCGCCCGTGTTGTCTGTGCCTTTATGGGACTCAGGAGACAGGAGGCGGGGGAGGCCCCGCAGGCCGGCCGGGGGCTGGGGGGCGGCCGCCGGGCATCTGGGCCTCACCCTCCCCTGGCAT

>KCNC4-Oorc-wPrimer

GGTGCTGCCGGTAGGTCATCCAGCAGCAGGGCTCCACGTCGGTCTCGTCGATGCCCCAGAAGGTGAGCTCCTCCTCAAACAGAGGCCCGCACACGTCGGCGGGGCAGTGCAGCTTGCCCGTGCGGTAGTAGTTGAGCACGTAAGCAAAGACGCCCGGGTGCCGGTCGAAGAAGAACTCGCAGCCACCGCCGCCGCTGCTGCCTGCACCACCGCCATCGGACGCGGGCCGGCCTCCGCCGTCGGGATCGGCCAGCCAGGCGAGGCGGGTGCCCGGTAGGGTGCGCAGGGTGCTGCGGTAGGTCTCATGTCGCGTGCCGCCCACGTTGATGATGATCTTCTCCGACGCCTCGCCCTTGGCCATCTCCTCCTTCAGACATGTTTTGGACGGAGG

>VGF-Oorc-wPrimer

CCAGTGGGCTGGGCTCAGCTGGATCCGCGCGGCTCCGGGAGACTCGCTCGCTCAGGCTTCAGCACGCTGGACAGCGCCCGCGCCGCTACCGCCTTATAAAGAGGAGCGCGCGGGGTCACGTGGGAATCGCCCCGCCCCATTGACGTCAATGTTCATTCATGGGGAAGCGGGCGGGCGCCTAGAGGCGGGAGGCCGCCCAGCGATTGGAGGATGCGTGCCTCTCCCGGCCCGCGCCTGCGCGCTGCAGGCCTCTTTGGAGGGGTGTGCGCTATCCTGGCACCAGGGAAATGAATGAATGAAGGAATGAATGAATGAAATGCTGAGGCGGGCGGGGCAGGGGGGCTATGAATGAATGAAGGAGGGACGAGGAGAAAAGGATGAATGAATGAATGGCAGAATG

>HMG20B-Oorc-wPrimer

CCTCTCAGAGCCCTCCTCTGAGGGAGGACTCAGGGCCCTCTCTGCCAACTCCGCCGTCGGTGTTGGGAAGTGGGGGTGACCCTGGCTTCCCATGGCTCCAGGCCGGCGAGCGGCAAGGCTCCAGGACAGCATGGGGGCTTCGTGGTGGCTGTCAAGCAAGAGCGCGGCGAGGGCCCGAGGGCCGGAGATAAGGGGTCCCACGAGGAGGAGGTGAGAGTCCCTGCACTGTGGTGGCGGCGGCCCTTAGTCTTGAAGCTCCGCCCCGAACTCAATTTGAATTCGTTGTGGCTCCGCCCACAGTCCAGATTTCCCTCCAGATGCTGAGGCCCCGCCC

>PRDM12-Oorc-wPrimer

AGGGGCCGCAGGGAGGAGGGGACGCGATCGGCCTCGACGGCGCGACTCACCGCCCGAGAAGGACTGCGCCAGGACCTCGGCGGTGAAGGCTGTCTTGGGGCTGGCGTGGTGGCTCTTGTCTTCGAAGAGCTGCTCGCCCAGCACGTTGCGCCAGCGGCCGTACAGGAAGCTGTGCAGGATGTCGGAGGTGATGACCTCTGCCAGCGCCAGCCCCGGCGCCTTCAGCCCTGTCTTGAGCACCAGGGCCTCAGCCGGGAGCACGGAGCCCATCATGGGCGGCCCGGGGCTCGCCGGCGCCGGGGACGGACGGCCGGACGGCCGGGCCGAGGCGGGTATGTGGTGGGCGAGTGGGGAGCACAGGGGCGAGAGAAGAGGGCTGTGATGGGGGAGGACGAGGA

False killer whale bisulfite-converted consensus sequences:

>TET2-Pcra-consensus

GTTGATGTTATTATTTAATTATAAAGTTGGATATTTTTTTAAAATAGAGGTTTTTATGGGGGTAAATTTAAGTATTTGAAAGTGTAGATTTATTTTTTATAATCGAATTTAGCGCGAAGTGGAGAGTTATTGGTGCGAAAGTTTTTTTGGATTTGTGTGAATTTTTTTTTTTAGTTTATGTGGTTAAAGTAAATAGAAGGTGGGTTGGGGTGGGGAGAAGTGGGTTTGGGTTAAATTTTTAATTTGTTGAGTTT

>GRIA2-Pcra-consensus

TGTATGGGAGGGTGTTGAGTGTGTTGAGGTGTTGGGATTATAGTGGTAGTTTGTTGGAAGTTGTATTTAGTTAGTTTTTGGATTTTGTGAGTGGGGATTGGGTGTAGGGTGGTAGTTATTTGTAGGATTTTGGAAATAGGGATTTTTTTGTTATTATGTTGGGTTTTTAGTAGTTTGGTGTTATATTGTTGTTTTAAAATGTAGAGGATTTAATTTGTTGAGGAGAATGGTTAAAGAAGGAAGAGGAGGAAAAGGGGGGAAAAAAAGGGTATTTTGTGGATGTTTTATTTTTTT

>DIRA-Pcra-consensus

TATTTGTTTGTTTTAGAGGAGGAGTAGAGATTTTCGCGTGGTGGTGTTCGGTTCGGTCGGCGTGGGTAAGAGCGCGTTGGTGTAGAGGTGGGTGCGCGGTAATTTTCGTGAGGCGTATTTGTCGATTATCGAAGATATTTATCGTTAGGCGTTGGGTTGTAGTTATAAGGCGGGCGTATTGTATATTATCGATATTATCGGCGGTCGTCGTTATCGGGGTTTGTAGCGTTTCGTTATTGTTAGGGGTTACGTTTTTATTTTGGTTTATTTCGTTATTAAGAAGTAAATTTTGGAGGAGTTGAAGTTTTTTTTTGAGTTGATTTGTTAA

>FLJ0945-Pcra-consensus

ATTTAATTTAAATATGGAAATGTTTAGGTTAGGTAATGTAATTATTTTTGAGGTTTGGTGGGTTGTGTAGGTTTTTGGTTTTGGGGAGTATGGTGGTATTGTTTTTTTTTTGGTGTGTGTGAAATTATGTGTATAAAAGGTGTTTTTGTATTTGAGTGTGTGAATTAAATAAATAAATAAGGGTTTTTGTTTGTTTGTTGGTTTTTGTATATGATTGTTTTGATTAAATTGTTTGTGTTGTTTGTTTTTATGGGATTTAGGAGATAGGAGGTGGGGGAGGTTTTGTAGGTTGGTTGGGGGTTGGGGGGTGGTTGTTGGGTATTTGGGTTTTATTT

>KCNC4-Pcra-consensus

TTGTTGGTAGGTTATTTAGTAGTAGGGTTTTACGTCGGTTTCGTCGATGTTTTAGAAGGTGAGTTTTTTTTTAAATAGAGGTTCGTATACGTCGGCGGGGTAGTGTAGTTTGTTCGTGCGGTAGTAGTTGAGTACGTAAGTAAAGACGTTCGGGTGTCGGTCGAAGAAGAATTCGTAGTTATCGTCGTCGTTGTTGTTTGTATTATTGTTATTGGATGTGGGTTGGTTTTTGTTGTTGGGATTGGTTAGTTAGGTGAGGTGGGTGTTTGGTAGGGTGTGTAGGGTGTTGTGGTAGGTTTTATGTTGTGTGTTGTTTATGTTGATGATGATTTTTTTTGATGTTTTGTTTTTGGTTATTTTTTTTTTTAGATATGTTTTGGAT

>VGF-Pcra-consensus

AGGGTTGGGTTTAGTTGGATTTGTGTGGTTTTGGGAGATTTGTTTGTTTAGGTTTTAGTATGTTGGATAGTGTTTGTGTTGTTATTGTTTTATAAAGAGGAGTGTGTGGGGTTATGTGGGAATTGTTTTGTTTTATTGATGTTAATGTTTATTTATGGGGAAGTGGGTGGGTGTTTAGAGGTGGGAGGTTGTTTAGTGATTGGAGGATGTGTGTTTTTTTTGGTTTGTGTTTGTGTGTTGTAGGTTTTTTTGGAGGGGTGTGTGTTATTTTGGTATTAGGGAAATGAATGAATGAAGGAATGAATGAATGAAATGTTGAGGTGGGTGGGGTAGGGGGGTTATGAATGAATGAAGGAGGGATGAGGAGAAAAGGATGAATGAATGAATGGTAG

>HMG20B-Pcra-consensus

AGTTTTTTTTTGAGGGAGGATTTAGGGTTTTTTTTGTTAATTTTGTTGTTGGTGTTGGGAAGTGGGGGTGATTTTGGTTTTTTATGGTTTTAGGTTGGTGAGTGGTAAGGTTTTAGGATAGTATGGGGGTTTTGTGGTGGTTGTTAAGTAAGAGTGTGGTGAGGGTTTGAGGGTTGGAGAGAAGGGGTTTTATGAGGAGGAGGTGAGAGTTTTTGTATTGTGGTGGTGGTGGTTTTTAGTTTTGAAGTTTTGTTTTGAATTTAATTTGAATTTGTTGTGGTTTTGTTTATAGTTTAGATTTTTTTTTAGATGTTGAGGTT

>PRDM12-Pcra-consensus

TTGTAGGGAGGAGGGGATGTGATTGGTTTTGATGGTGTGATTTATTGTTTGAGAAGGATTGTGTTAGGATTTTGGTGGTGAAGGTTGTTTTGGGGTTGGTGTGGTGGTTTTTGTTTTTGAAGAGTTGTTTGTTTAGTATGTTGTGTTAGTGGTTGTATAGGAAGTTGTGTAGGATGTTGGAGGTGATGATTTTTGTTAGTGTTAGTTTTGGTGTTTTTAGTTTTGTTTTGAGTATTAGGGTTTTAGTTGGGAGTATGGAGTTTATTATGGGTGGTTTGGGGTTTGTTGGTGTTGGGGATGGATGGTTGGATGGTTGGGTTGAGGTGGGTATGTGGTGGGTGGGTGGGGAGTATAGGGGTGAGAGAAGAGGGTTGTGATGGGGGAGGAT
