# Supplementary material for: Epigenetic Age Estimation for Hawaiian False Killer Whales (Pseudorca crassidens) in the Absence of ‘Known‐Age’ Individuals
Source: Mol Ecol Resour. 2026 Jan 17;26(2):e70099. doi: 10.1111/1755-0998.70099 (PMC12811820; doi:10.1111/1755-0998.70099)
Supplement: Supplementary file 1 — Data S1: men70099‐sup‐0001‐DataS1.zip. Table S1: Primer design and optimization details for the 12 loci for which we designed primers. The first eight loci were used in this study, while the remaining four were eliminated following optimisation. Start and end positions and sequence length refer to the total sequence extracted from the O. orca genome for use in primer design, while amplicon length is length of the product amplified by the primers we designed. The final column lists the number of mutations between the O. orca sequence and the false killer whale consensus sequence generated from our data (for loci retained in study) or optimisation notes (for loci omitted). Table S2: Predictive accuracy of all models (n = 318). The MAE and mean residual are given for all samples, as well as for those with Agebest estimates between 0 and 9 years, 10 to 24 years, and 25 to 40 years. Corr is the Pearson's correlation coefficient between Agebest and predicted age. Models are sorted by overall MAE. Figure S1: The difference between Agemax and Agemin as a function of Agebest. Points are colour‐coded based on their confidence rating. Note that, for a given age class, the range of plausible ages for high‐confidence samples (confidence ≥ 4) is consistently smaller than for low‐confidence samples. Figure S2: Median number of reads per sample (A) and per CpG site (B) in the final data set. Figure S3: Heat maps showing absolute value of correlation coefficients of methylation among CpG sites within a locus. Figure S5: Predicted age probability distributions from the false killer whale age clock for samples with confidence ratings of 3. Each panel shows results for a different individual. The solid black lines show the lower and upper limits of the 95% high‐density interval (HDI) of the distribution. Values outside of the HDI are shown in grey bars. The dotted grey line is at Agebest, while the solid grey lines show the Skew‐Normal age probability distribution from Kratofil et al. [file MEN-26-e70099-s001.zip › men70099-sup-0001-DataS1/men70099-sup-0003-FigureS4.pdf]

MOLECULAR ECOLOGY

Figure S4. Plots of *Age*$_{best}$ versus the probability of methylation ($\widehat{P_m}$) for all 184 CpG sites included in the final data set. Each plot is labeled with [locus name]_[position]. The line of best fit, as calculated using the R function *geom_smooth(method = 'lm'), is shown in blue.*

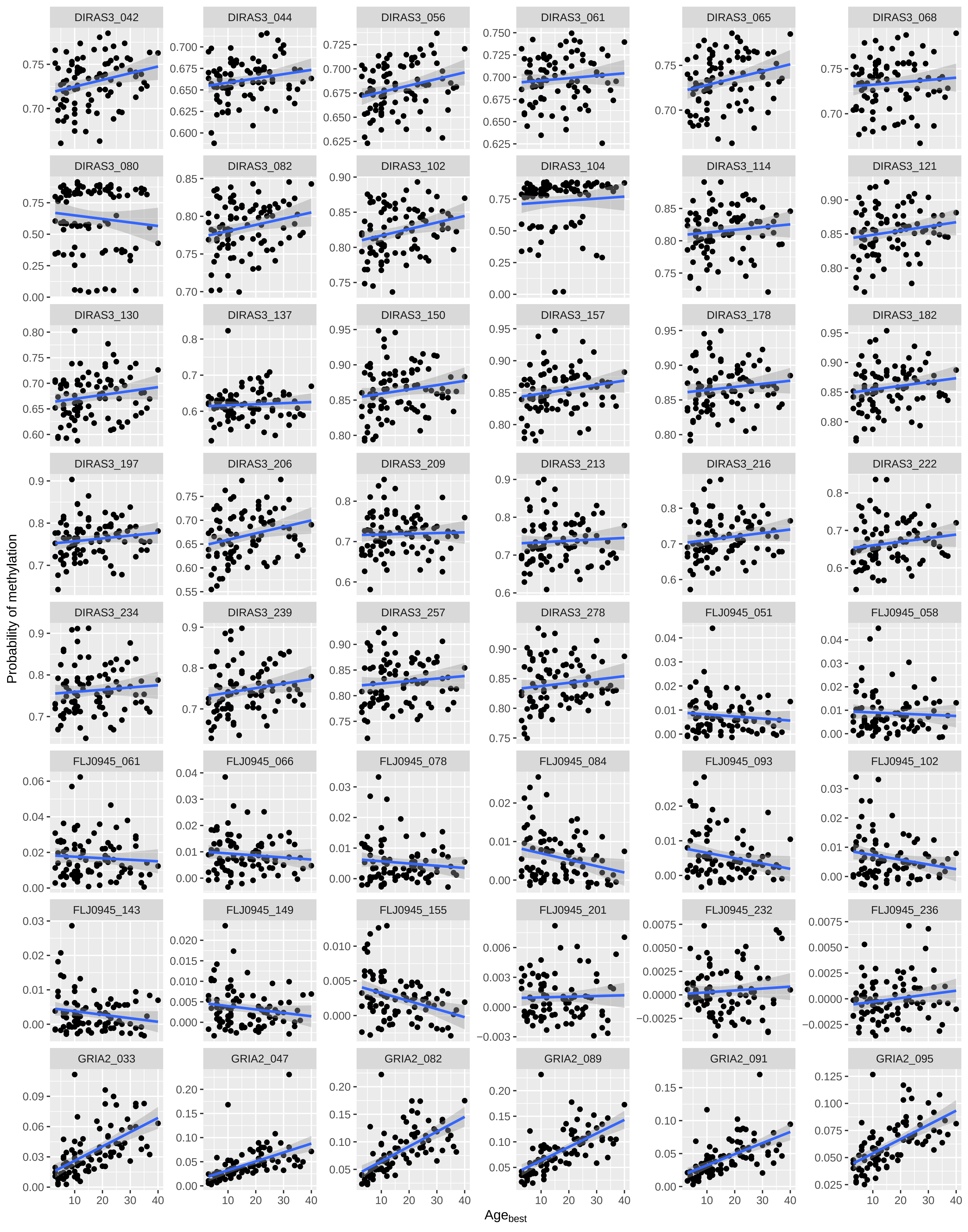
DIRAS3_042
DIRAS3_044
DIRAS3_056
DIRAS3_061
DIRAS3_065
DIRAS3_068
DIRAS3_080
DIRAS3_082
DIRAS3_102
DIRAS3_104
DIRAS3_114
DIRAS3_121
DIRAS3_130
DIRAS3_137
DIRAS3_150
DIRAS3_157
DIRAS3_178
DIRAS3_182
DIRAS3_197
DIRAS3_206
DIRAS3_209
DIRAS3_213
DIRAS3_216
DIRAS3_222
DIRAS3_234
DIRAS3_239
DIRAS3_257
DIRAS3_278
FLJ0945_051
FLJ0945_058
FLJ0945_061
FLJ0945_066
FLJ0945_078
FLJ0945_084
FLJ0945_093
FLJ0945_102
FLJ0945_143
FLJ0945_149
FLJ0945_155
FLJ0945_201
FLJ0945_232
FLJ0945_236
GRIA2_033
GRIA2_047
GRIA2_082
GRIA2_089
GRIA2_091
GRIA2_095
Probability of methylation
Age$_{best}$

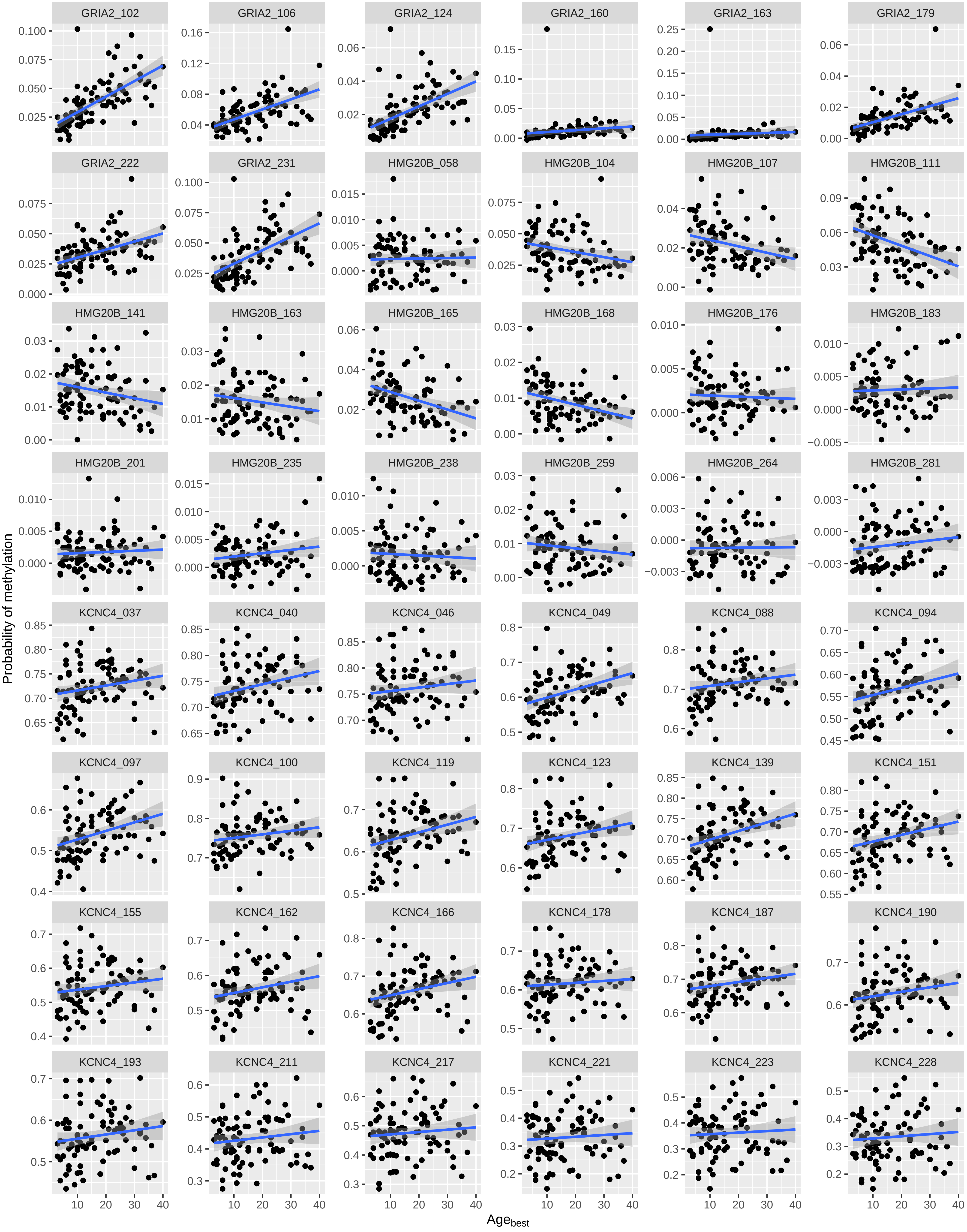

GRIA2_102
GRIA2_106
GRIA2_124
GRIA2_160
GRIA2_163
GRIA2_179
GRIA2_222
GRIA2_231
HMG20B_058
HMG20B_104
HMG20B_107
HMG20B_111
HMG20B_141
HMG20B_163
HMG20B_165
HMG20B_168
HMG20B_176
HMG20B_183
HMG20B_201
HMG20B_235
HMG20B_238
HMG20B_259
HMG20B_264
HMG20B_281
KCNC4_037
KCNC4_040
KCNC4_046
KCNC4_049
KCNC4_088
KCNC4_094
KCNC4_097
KCNC4_100
KCNC4_119
KCNC4_123
KCNC4_139
KCNC4_151
KCNC4_155
KCNC4_162
KCNC4_166
KCNC4_178
KCNC4_187
KCNC4_190
KCNC4_193
KCNC4_211
KCNC4_217
KCNC4_221
KCNC4_223
KCNC4_228
Probability of methylation
Age_{best}

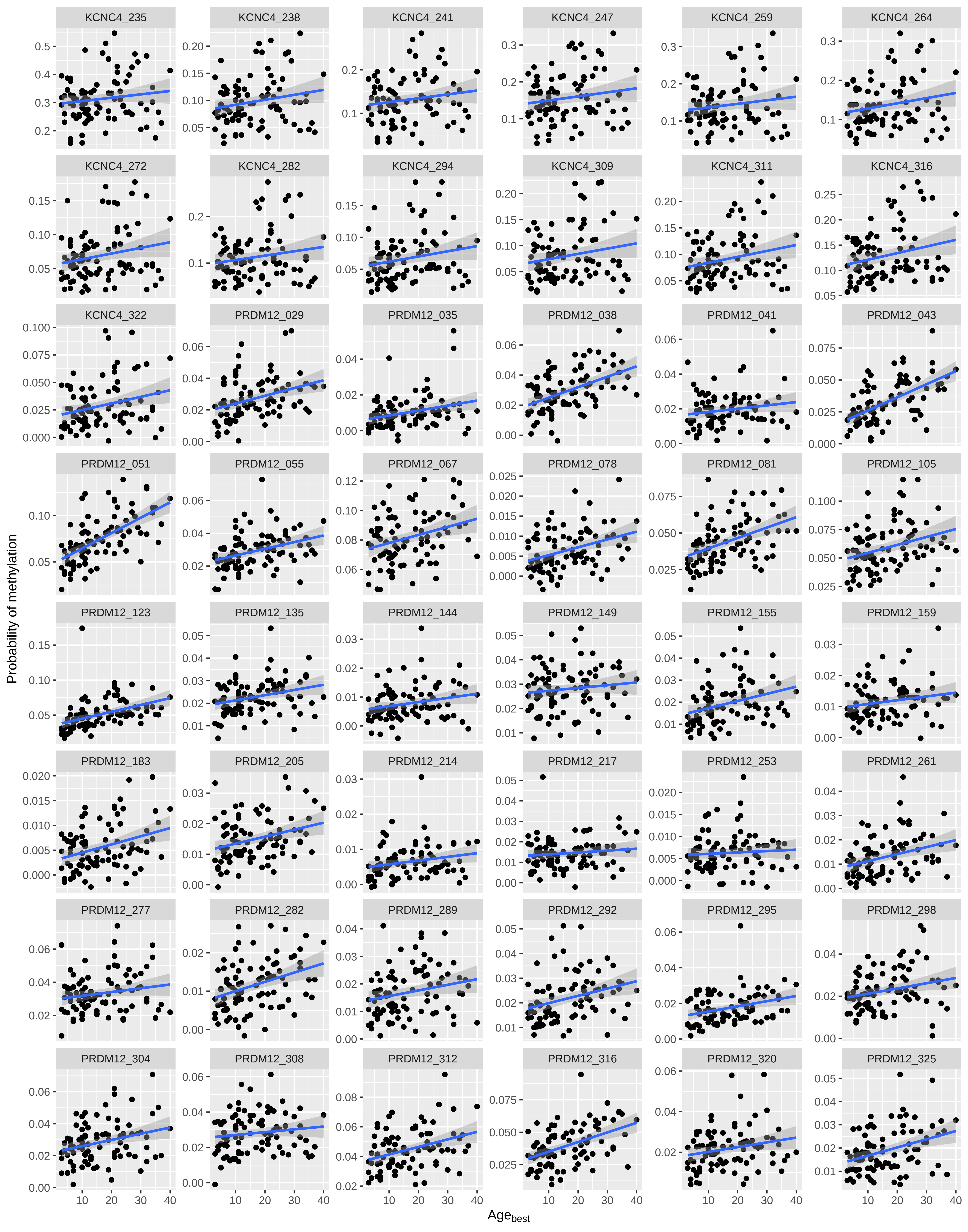
KCNC4_235
KCNC4_238
KCNC4_241
KCNC4_247
KCNC4_259
KCNC4_264
KCNC4_272
KCNC4_282
KCNC4_294
KCNC4_309
KCNC4_311
KCNC4_316
KCNC4_322
PRDM12_029
PRDM12_035
PRDM12_038
PRDM12_041
PRDM12_043
PRDM12_051
PRDM12_055
PRDM12_067
PRDM12_078
PRDM12_081
PRDM12_105
PRDM12_123
PRDM12_135
PRDM12_144
PRDM12_149
PRDM12_155
PRDM12_159
PRDM12_183
PRDM12_205
PRDM12_214
PRDM12_217
PRDM12_253
PRDM12_261
PRDM12_277
PRDM12_282
PRDM12_289
PRDM12_292
PRDM12_295
PRDM12_298
PRDM12_304
PRDM12_308
PRDM12_312
PRDM12_316
PRDM12_320
PRDM12_325
Probability of methylation
Age best

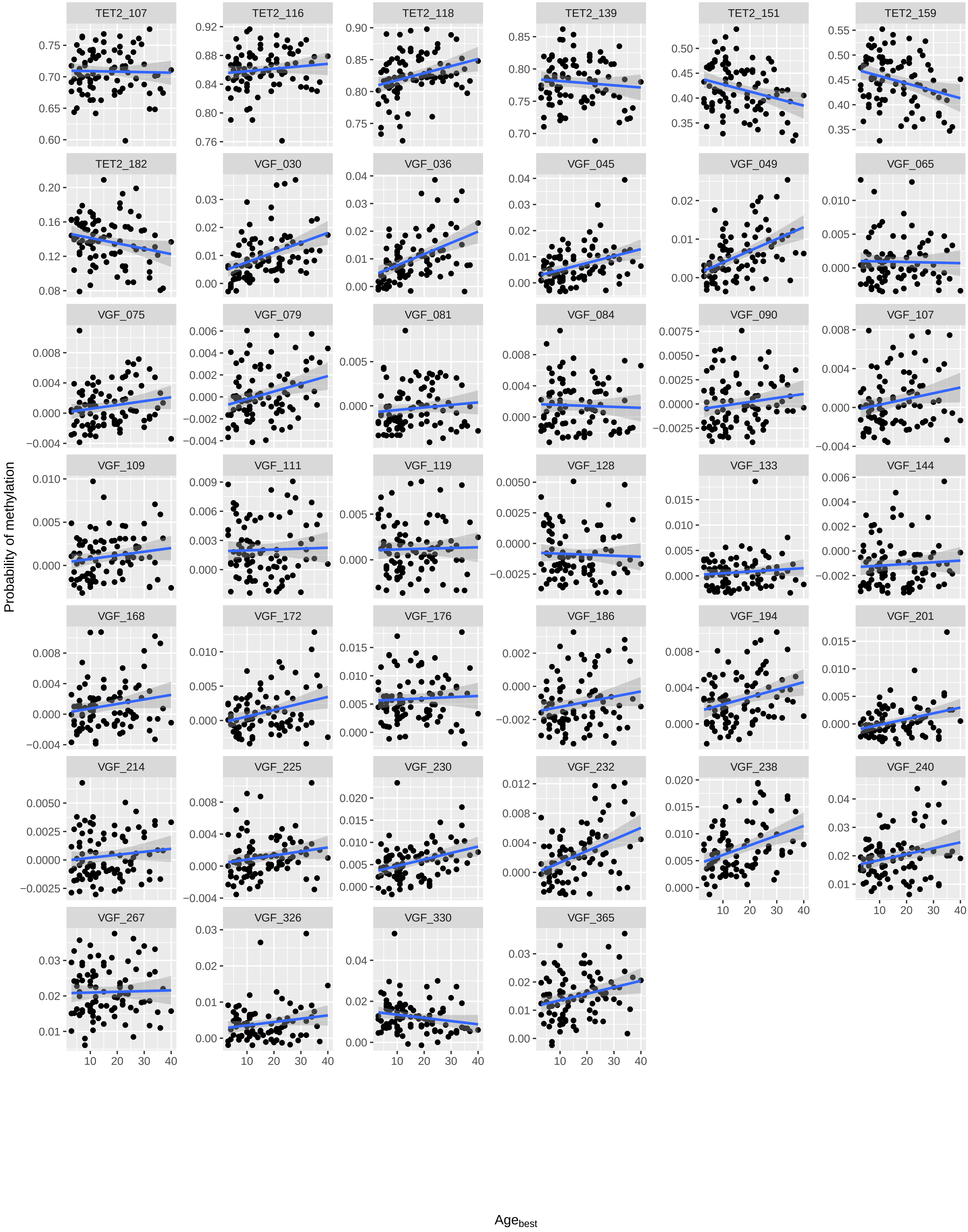

TET2_107
TET2_116
TET2_118
TET2_139
TET2_151
TET2_159
TET2_182
VGF_030
VGF_036
VGF_045
VGF_049
VGF_065
VGF_075
VGF_079
VGF_081
VGF_084
VGF_090
VGF_107
VGF_109
VGF_111
VGF_119
VGF_128
VGF_133
VGF_144
VGF_168
VGF_172
VGF_176
VGF_186
VGF_194
VGF_201
VGF_214
VGF_225
VGF_230
VGF_232
VGF_238
VGF_240
VGF_267
VGF_326
VGF_330
VGF_365
Probability of methylation
Age$_{best}$
